# Co-rotating twin screw process for continuous manufacturing of solid crystal suspension: A promising strategy to enhance the solubility, permeation and oral bioavailability of Carvedilol

**DOI:** 10.12688/f1000research.139228.3

**Published:** 2024-05-22

**Authors:** Prerana D. Navti, Gasper Fernandes, Soji Soman, Ajinkya N. Nikam, Sanjay Kulkarni, Sumit R Birangal, Namdev Dhas, Gautham G. Shenoy, Vinay Rao, Kunnatur Balasundara Koteshwara, Srinivas Mutalik

**Affiliations:** 1 Department of Pharmaceutics, Manipal College of Pharmaceutical Sciences, Manipal Academy of Higher Education, Manipal, Karnataka, 576104, India; 2 Department of Pharmaceutical Chemistry, Manipal College of Pharmaceutical Sciences, Manipal Academy of Higher Education, Manipal, Karnataka, 576104, India; 3 STEERLife, Steer Engineering Pvt Ltd, No. 290, 4th Main Road, Ganapathy Nagar, Phase 3, Peenya Industrial Area, Peenya, Bengalore, Karnataka, 560058, India

**Keywords:** Twin screw processor, solid crystal suspension, carvedilol, mannitol, solubility, in vitro dissolution, ex vivo permeation, in vivo pharmacokinetics

## Abstract

**Background:**

In the current work, co-rotating twin-screw processor (TSP) was utilized to formulate solid crystal suspension (SCS) of carvedilol (CAR) for enhancing its solubility, dissolution rate, permeation and bioavailability using mannitol as a hydrophilic carrier.

**Methods:**

*In-silico* molecular dynamics (MD) studies were done to simulate the interaction of CAR with mannitol at different kneading zone temperatures (KZT). Based on these studies, the optimal CAR: mannitol ratios and the kneading zone temperatures for CAR solubility enhancement were assessed. The CAR-SCS was optimized utilizing Design-of-Experiments (DoE) methodology using the Box-Behnken design. Saturation solubility studies and *in vitro* dissolution studies were performed for all the formulations. Physicochemical characterization was performed using differential scanning calorimetry , Fourier transform infrared spectroscopy, X-ray diffraction studies, and Raman spectroscopy analysis. *Ex vivo* permeation studies and *in vivo* pharmacokinetic studies for the CAR-SCS were performed. Stability studies were performed for the DoE-optimized CAR-SCS at accelerated stability conditions at 40 ºC/ 75% RH for three months.

**Results:**

Experimentally, the formulation with CAR: mannitol ratio of 20:80, prepared using a KZT of 120 ºC at 100 rpm screw speed showed the highest solubility enhancement accounting for 50-fold compared to the plain CAR. Physicochemical characterization confirmed the crystalline state of DoE-optimized CAR-SCS. *In-vitro* dissolution studies indicated a 6.03-fold and 3.40-fold enhancement in the dissolution rate of optimized CAR-SCS in pH 1.2 HCl solution and phosphate buffer pH 6.8, respectively, as compared to the pure CAR. The enhanced efficacy of the optimized CAR-SCS was indicated in the *ex vivo* and *in vivo* pharmacokinetic studies wherein the apparent permeability was enhanced 1.84-fold and bioavailability enhanced 1.50-folds compared to the plain CAR. The stability studies showed good stability concerning the drug content.

**Conclusions:**

TSP technology could be utilized to enhance the solubility, bioavailability and permeation of poor soluble CAR by preparing the SCS.

## Introduction

The bioavailability of drugs *via* the oral route majorly depends on the drug solubility and the dissolution rate. Solubility acts as a rate-determining step for the effective therapeutic response of the drug. ^1^ Specifically, the drugs belonging to the biopharmaceutical classification system (BCS) class II suffer from poor absorption and low bioavailability. Therefore, there is a concern about increasing the solubility of these drug candidates. ^2^ Many approaches like co-solvency, surfactants, micronization, complexation, hydrotropy, salt formation, cocrystals, and co-amorphous technology have been extensively utilized for the solubility enhancement. ^1^^,^^3^^,^^4^

A new theory known as solid crystal suspensions (SCS) has recently been proposed wherein the ground drug is homogeneously distributed in the crystalline carrier matrix using hot-melt extrusion (HME) without aiding in any drug and the crystalline matrix interaction, forming a stable product having a better dissolution rate compared to the bulk drug. ^5^ Highly hydrophilic carriers enhance the drug solubility in the SCS. The enhanced wetting and drug particle size reduction improve the dissolution and solubility of the poorly soluble drug. ^5^ The enhancement in the solubility due to the particle size reduction as described by Noyes Whitney Equation is due to the increased surface area to volume ratio as the particle size decreases. The increased surface area facilitates higher interaction with the solvent molecules resulting in increased solubility. ^6^^,^^7^ A solid crystal system differs from that of amorphous solid dispersions, ^4^^,^^5^^,^^8^ which are thermodynamically unstable because of their ability to get converted to a more stable crystalline form. Also, moisture absorption for the amorphous forms is more severe than the crystalline state and results in agglomeration of the amorphous form. Since no amorphous phase is involved, the physical stability of SCS is higher.

Carvedilol is an alpha and beta-blocker utilized in hypertension treatment belonging to BCS class II with high permeability and low solubility. CAR exists as a white crystalline powder with rod shaped particles. It is practically insoluble in acidic solvents and water but displays slight solubility in ethanol and ether. It is soluble in organic solvents like methylene chloride and methanol and is freely soluble in dimethylsulfoxide. Solubility of CAR depends upon the pH of the solvent. The solubility ranges from <1 μg/mL at a pH higher than 9.0, 100 μg/mL at a pH of 5.0 and 23 μg/mL at a pH of 7.0 at a room temperature. ^9^^,^^10^ The bioavailability of carvedilol is very low orally, which accounts for only 25%. Hence, there is an immense need to enhance its solubility and improve bioavailability. ^3^^,^^11^^,^^12^ The approaches utilized for solubility and dissolution rate improvement of carvedilol include micronization, ^7^ solid dispersion, ^11^^,^^13^ cyclodextrin inclusion complex, ^14^^–^^16^ cocrystallization, ^3^^,^^17^ co-amorphous technology ^18^ and nanotechnology. ^12^ To date, the SCS approach has not been explored for solubility enhancement of carvedilol.

HME technology has previously been reported for the preparing SCS of several drugs such as efavirenz, ^5^ griseofulvin, ^8^ and griseofulvin, phenytoin, and spironolactone. ^4^ However, the HME machines reported in earlier studies for SCS preparation consisted of a single barrel with only one heating zone in which temperature variation is quiet difficult. ^4^^,^^5^^,^^8^ There are no reports on the application of twin-screw processor (TSP) technology using different heating zones in the preparation of SCS. Twin screw processing refers to directing the raw materials in the machine consisting of one or two rotating screws wherein the starting materials are exposed to different temperatures, followed by passing the material via the die to give the product. ^19^^,^^20^ The fed material creates a melt pool inside the melting zone of the extruder facilitating the interaction and homogenous mixing between the raw materials. ^18^ This technology has been used for the solubility/dissolution enhancement of poorly soluble drugs by crystal engineering, cyclodextrin complexation and by cocrystal manufacture. ^18^ Different heating zones in the barrel allow heat distribution, heat optimization, and adaptation of the processing section for the product. ^21^ Because of this reason, altering the temperature in a barrel is essential. Typically, the temperature in the melting and kneading zone is greater compared to the conveying zone. ^21^ In the present work, we utilized a co-rotating TSP instrument having different heating zones in contrast to above listed previous reports.

The goal of this work was to enhance the oral bioavailability of the BCS Class II drug, carvedilol by exploring the theory of SCS using a water-soluble crystalline carrier by employing TSP technology. The polyols (sorbitol and mannitol) were explored as crystalline carriers in the current study for SCS formation. Mannitol was screened as the main crystalline carrier owing to the higher enhancement in the solubility of the CAR in the crystalline suspension obtained with mannitol compared to sorbitol in the trials taken. The SCS of carvedilol using mannitol was prepared and evaluated by utilizing Design of experiments (DoE) methodology. Box-Behnken design (BBD) was utilized for the optimization wherein kneading zone temperature, screw speed, and CAR:mannitol ratio were selected as the independent factors, and solubility was the response. In addition, the aspects of *in-silico* molecular dynamics (MD) were also applied to study the interactions between the CAR and mannitol in different ratios of CAR: mannitol (20:80, 35:65, and 50:50) employed in this work at various kneading zone temperatures (120°C, 130°C, and 140°C) to predict the optimal ratios for preparing CAR-SCS formulation which would yield higher solubility, on the basis of hydrophilicity and hydrophobicity report for all the formulated SCS formulations of CAR generated from *in-silico* MD studies. There are no reports available on the use of such *in-silico* MD studies for the preparation of SCS for better solubility.

The CAR:mannitol ratios and the kneading zone temperature for the optimized solution obtained from the Design Expert software was similar to one amongst the four best solutions suggested by the *in-silico* MD studies. Also, the stability of the optimum CAR-SCS with CAR:mannitol ratio of 20:80, and a kneading zone temperature of 120°C was assessed using *in-silico* MD studies. The DoE optimized CAR-SCS (F8) was characterized for different physicochemical properties *i.e.* differential scanning calorimetry (DSC), Fourier transform infrared spectroscopy (FTIR), powder X-ray Diffraction (P-XRD) and nuclear magnetic resonance (NMR) analysis. The DoE optimized CAR-SCS (F8) was characterized for different physicochemical properties i.e. differential scanning calorimetry (DSC), Fourier transform infrared spectroscopy (FTIR), powder X-ray Diffraction (P-XRD), nuclear magnetic resonance, Raman spectrometric analysis, thermogravimetry analysis (TGA), scanning electron microscopy (SEM), Energy Dispersive spectroscopy (EDS) and nuclear magnetic resonance (NMR) analysis. The CAR-SCS (F8) was subjected to *ex vivo* permeation and *in vivo* pharmacokinetic (PK) study to understand the dissolution and absorption rate of CAR.

## Methods

### Materials

CAR was obtained as a gift sample from Zydus Cadila Healthcare Ltd, Kundaim, Goa, India. Mannitol and sorbitol were purchased from Universal Laboratories Private Ltd, Mumbai, India. Methanol, acetonitrile (ACN), Triethylamine (TEA) and ortho-phosphoric acid (OPA) were obtained from Loba Chemie Pvt Ltd (Mumbai, India). 0.22 μ membrane filters were obtained from Chemixol agencies, Mangaluru. All the chemicals and solvents utilized were of analytical grade.

### Selection of crystalline polyol for the preparation of solid crystal suspension of carvedilol

A TSP instrument (O-Micron 10P, Steer Engineering, Bangalore, India) with co-rotating twin screws having an outer to the inner diameter ratio (Do/Di) of 1.71 were utilized for the study. The TSP barrel consisted of four zones (B1, B2, B3 and B4). The screw configuration consisted of four processing segments throughout the barrel zones for facilitating thorough mixing of the contents. First screw section in B1 was conveying zone with minimum mixing capability. Second screw segment in B2 was a mixing zone to facilitate good mixing. This was followed by a third screw section (kneading zone in B3) to facilitate melting along with homogenous mixing followed by the discharge end of the screws (in B4 zone). A few initial trials were taken using sorbitol and mannitol as hydrophilic carriers by preparing a 50:50 mixture using mortar and pestle. The TSP barrels B1, B2 and B4 were set at 31°C, 100°C, 95°C respectively. The B3 (kneading zone) temperature range was selected as 70-110°C for sorbitol and 120-140°C for mannitol, considering the melting point of the crystalline polyols, and the screw rpm was varied from 50-150 rpm ( Table 1). From the results obtained by solubility studies for both the polyols, mannitol was screened as the primary hydrophilic carrier for formulating the CAR-SCS using DoE ( Table 1).

**Table 1.** Preliminary trials conducted using sorbitol and mannitol as the hydrophilic carriers.

Hydrophilic carrier used	Drug:Hydrophilic carrier ratio	Kneading zone temperature (°C)	Screw rpm	Solubility (mg/mL)
Mannitol	50:50	120	50	0.548
		120	75	0.650
		130	50	0.820
		130	100	0.887
		130	150	1.003
		140	50	0.382
		140	100	0.400
Sorbitol	50:50	80	50	0.310
		80	100	0.538
		110	50	0.420
		110	100	0.822

### Formulation of Solid crystal suspension of Carvedilol (CAR-SCS) by TSP using BBD

BBD was utilized to systematically evaluate the effect of TSP instrument parameters like kneading zone temperature, screw rpm, and formulation-related parameter (CAR:mannitol ratio) on the solubility of CAR from SCS formulations (response). The experimental runs and data analysis were performed utilizing Design-Expert software (Version 9.0.3.1) ( Chemoface is a free alternative software that may be able to perform similar functions). ANOVA was utilized to find the significant effect of the factors on response regression coefficients.

CAR and mannitol physical mixtures at the ratios of 50:50, 35:65, and 20:80 were prepared, blended using mortar and pestle, and extruded from the TSP die. The kneading zone temperature range of 120-140°C was used. The TSP zones B1, B2 and B4 were set at 32°C, 100°C and 95°C respectively. Zone B3 (kneading zone) temperature and screw rpm were set as per the runs suggested by the Design-Expert software ( Table 2).

**Table 2.** Carvedilol-solid crystal suspension formulation trials generated by the Design-Expert software.

Serial. No.	Formulation code	Drug:mannitol ratio	Kneading zone temperature (° C)	Screw rpm	Solubility (mg/mL)
1	F1	50:50	140	100	0.40
2	F2	35:65	140	150	0.69
3	F3	20:80	130	150	0.95
4	F4	35:65	140	50	0.51
5	F5	50:50	130	150	0.95
6	F6	35:65	130	100	0.90
7	F7	35:65	120	150	2.30
8	F8	20:80	120	100	2.50
9	F9	35:65	130	100	0.89
10	F10	20:80	140	100	0.73
11	F11	50:50	130	50	0.65
12	F12	35:65	120	50	1.60
13	F13	50:50	120	100	1.08
14	F14	20:80	130	50	0.84
15	F15	35:65	130	100	0.90

### *In-silico* studies for CAR-SCS

The molecular dynamics (MD) simulation studies of CAR with mannitol at different ratios, *i.e.*, 50:50, 35:65, and 20:80 with different kneading zone temperatures, were performed using Schrodinger software Suite (version 2023-2) (Schrodinger LLC, New York) in the Maestro module (version 13.6.121, MMShareVersion 6.2.121, Release 2023-2, Platform Linux-x86_64) (An alternative free software able to perform similar tasks is Gromacs RRID:SCR_014565). The guest molecule CAR and mannitol structures were built using the 2D sketcher and were optimized with LigPrep by using default settings at pH 7.0 coupled with Epik calculations. The optimization of the geometry of the CAR-SCS in the various ratios of CAR with mannitol ( *i.e.* 50:50, 35:65, and 20:80) was individually done utilizing the Macro Model minimization tool by keeping default settings.

### Molecular structure preparation using Disordered System Builder (DSB)

The two-dimensional structure of CAR and mannitol was built in the Maestro structure builder by referring to the Pubchem database ( PubChem CID: 2585) ^52^ and ( PubChem CID: 6251). ^53^ The optimization of the structures was done by employing OPLS4 forcefield LigPrep/Epik. ^22^

CAR and mannitol were selected in DSB. In the components setting, 50 molecules of CAR with mannitol were prepared in the ratios: 20:80, 35:65, and 50:50. A new orthorhombic periodic boundary condition with dimension 40 Å was created. Then OPLS4 force field was used to build the CAR-SCS, and all other settings were kept on default. The formed CAR-SCS was taken for MD simulation studies.

### Molecular simulation studies and stability determination

The Desmond Module (Version 22.4) of Schrodinger was utilized for running the MD simulations. The MD was conducted for 0, 25, 75, and 100 ns at different kneading zone temperatures used, *i.e.*, 395.15K (120°C), 403.15K (130°C), and 413.15K (140°C) with 1.013 bar pressure kept by utilizing the Nose Hoover Chain thermostat and Matrtyna-Tobias-Klein barostate. For studying the stability of the CAR-SCS, CAR binding mode to mannitol in the CAR-SCS at different CAR and mannitol ratios, *i.e.*, 20:80, 35:65, and 50:50 at different kneading zone temperatures, *i.e.*, 393.15 K (120°C), 403.15 K (130°C), 413.15 K (140°C), pressure (bar) 1.01325 and for 100 ns simulation time period was predicted; thus generating 1000 structural frames of SCS formulation which were saved in trajectory. The MD simulation was run on CAR and mannitol in the SCS. The trajectory generated after the simulations was utilized for calculating the root mean square deviation (RMSD) to show the stability of the optimal CAR-SCS formulation.

### Percentage yield

The CAR-SCS formulations were collected, and the practical yield (%) was found out employing the formula ^3^:

$$\%\text{yield}=\left(\text{Practical yield}/\text{theoretical yield}\right)\times 100$$

### Differential scanning calorimetry (DSC)

DSC analysis was done utilizing DSC-60 Plus with TA-60WS thermal analyzer (Shimadzu Corporation, Kyoto, Japan) for pure CAR, mannitol, physical mixture (PM) of CAR and mannitol at 1:1 ratio, and the DoE optimized CAR-SCS (F8), for the analysis of solid-state. ^20^ A 5 mg sample was enclosed in an aluminum pan and sealed. An empty pan was utilized as a blank. The heating of the samples was achieved at a of 10°C/min rate from room temperature to 300°C in a nitrogen environment at a 10 mL/min flow rate. The DSC thermograms were recorded for the individual samples. ^50^

### Thermogravimetry analysis (TGA)

TGA was performed to evaluate the thermal stability of CAR, PM, and CAR-SCS (F8). The analysis was done using DTA-TG device (DTG-60H, Shimadzu Co., Japan). About 4 mg of the sample was heated between 25-800°C at a 10°C/min heating rate under dynamic nitrogen atmosphere. Experiments were done at the flow rate of 50 mL/min.

### Fourier transform infrared (FTIR) spectroscopy

FTIR spectra were recorded for pure CAR, mannitol, 1:1 PM of CAR and mannitol, and DoE optimized CAR-SCS (F8) using Alpha II, ECO-ATR (Bruker, Germany) at the wavelength range of 4000-900 cm ^-1^ for investigation of interactions of the CAR and the mannitol. FTIR spectra was also recorded for the DoE-optimized F8 formulation. The powdered sample was placed on the stage, and a focused image was obtained. The images were obtained using OPUS 8.0 software (provided with the equipment used).

### Powder X-ray diffraction (XRD) studies

XRD was utilized to estimate the CAR’s physical form in the CAR-SCS (F8) formulation. The instrument used was Malvern PANalytical, Netherlands, with Empyrean 3 ^rd^ generation model. X-ray diffraction was carried out for CAR, mannitol, CAR-mannitol physical mixture (ratio: 1:1), and optimized CAR-SCS (F8) formulation with a 40 kV voltage and 15 mA tube current at 7-50° (2θ) range. ^50^

### Scanning electron microscopy (SEM) and energy dispersive spectroscopy (EDS)

EVO MA18 with Oxford EDS (Zeiss, Germany) scanning electron microscope was utilized for assessing the surface morphology of plain CAR, plain mannitol, and optimized CAR-SCS (F8). The samples were placed onto the aluminum stub employing double-sided adhesive tape, and a vacuum at ten torr was applied. The samples were then scanned with an electron beam and SEM images were taken. Samples were tested utilizing both SEM and EDS.

### Raman spectrometric analysis

Raman spectrometric analysis was performed for the CAR, mannitol, and optimized CAR-SCS (F8) formulation using I-Raman Plus (B&W TEK, Plainsboro, NJ, USA) fitted with an Ar-Ne instrument. For all the experiments, the excitation wavelength of 785 nm of 35 mW power was utilized, and the integration time was 10 sec.

### Nuclear magnetic resonance (NMR) analysis

Proton NMR ( ^1^H) was done for CAR, mannitol, and the CAR-SCS (F8) by dissolving an appropriate amount of the samples separately in dimethyl sulfoxide, and the analysis was performed using a 400 MHz-Bruker ASCEND TM 400 NMR analyzer (Billerica, MA, USA).

### Saturation solubility determination

The saturation solubility of plain CAR and all the formulations was assessed in pH 6.8 phosphate buffer. Excess amounts of CAR and CAR-SCS formulations were separately incorporated in 2 mL of pH 6.8 phosphate buffer in the Eppendorf tubes, and the tubes were kept in a tube rotator (Neuation, *i* Roll PR35, Gandhinagar, Gujarat, India) for 48 h at 50 rpm speed. Later, the solutions were centrifuged at 10,000 rpm for 7 min. The supernatant was appropriately diluted and analyzed by a UV spectrophotometer at 241 nm.

### Determination of flow properties

The micrometric properties of CAR and optimized CAR-SCS (F8), including tap density, angle of repose, bulk density, and Hausner’s ratio, were evaluated. Tap density and compressibility index were assessed by the tapping method. Tap density was estimated using tap density tester (Electrolab ETD-1020, Mumbai, Maharashtra, India). The CAR and CAR-SCS was transferred using a funnel to a graduated 100 mL cylinder. The weight was determined using weighing balance (Wensar, Bengaluru, India) and the volume was recorded visually for the bulk density determination. The cylinder was tapped 1000 times and tap density calculation was done according to the formula below. ^23^^–^^26^

$$\text{Bulk density}=\frac{\text{mass}}{\text{bulk volume}}$$

$$\mathrm{Tap}\;\text{density}=\frac{\text{mass}}{\text{tapped volume}}$$

Hausner’s ratio is the ratio of tap density to the bulk density. Carr’s index (CI) is a measure of particle size, flow rate and cohesiveness. It was calculated using the formula:

$$\mathrm{CI}=\frac{\left(\mathrm{Tap}\;\text{density}-\text{bulk density}\right)}{\text{Tapped density}}\times 100$$

The fixed funnel methodology was utilized for measuring the angle of repose ^3^ wherein a funnel was fixed onto a stand such that the funnel tip was 2.5 cm above the flat surface on which a graph paper was placed. The powders were allowed to freely fall until the tip of the heap touched the funnel. The radius and height of the heap was measured. The formula utilized for the calculation was as follows ^24^^,^^27^:

$$Ɵ=\tan^{-1}\;\frac{\text{Pile height}}{\text{Pile radius}}$$

### Drug content

10 mg CAR-SCS were dissolved in little amount of methanol, and diluted using 0.1N HCl to 100 mL. This solution was sonicated using an ultrasonic bath (Antech,GT sonic, Panacea Instruments Pvt Ltd, New Delhi, Delhi, India) for 10 min. After diluting 0.1 mL of the solution to 1 mL with 0.1N HCl, it was filtered and analyzed using UV spectrophotometer (UV-1800 UV/Vis spectrophotometer, Shimadzu, Kyoto, Japan) at 241 nm ^3^.

### *In vitro* dissolution study

Dissolution studies were done utilizing USP type II dissolution apparatus (Electrolab TDT-08L Dissolution Tester) for CAR and all the CAR-SCS separately in both pH 1.2 HCl solution and phosphate buffer pH 6.8. Sodium lauryl sulfate (SLS) at 0.1% concentration was incorporated in both the dissolution solutions. A quantity of CAR and all CAR-SCS corresponding to 6.25 mg of CAR was loaded into the capsules of Size 4, and the capsules were placed in the 500 mL of dissolution medium maintained at 37 ± 0.5°C with a 50-rpm paddle speed. 5mL of samples were taken at 0.25 h, 0.5 h, 0.75 h, 1 h, and 2 h, and the equivalent fresh medium was incorporated into the dissolution jar. The collected samples were filtered using 0.45 micron filter and were analyzed by utilizing a UV spectrophotometer at 241 nm. ^3^^,^^28^

### Laboratory animals

Eighteen healthy male Wistar rats (eight weeks old) with body weight of 200-250 g were utilized for this study. The *ex vivo* study (n=3 per group/experiment with two groups) and *in vivo* pharmacokinetic study protocol (n=4 per group with three groups) was approved by Institutional Animal Ethics committee, Kasturba Medical College, Manipal (IAEC Registration No.: 94/PO/RReBi/S/99/CPCSEA). The number of rats per group for the respective experiments was decided based upon the existing literature. ^20^^,^^29^^,^^30^ Each group consisted of four animals maintained in a cage for the *in vivo* pharmacokinetic studies and three animals per cage for *ex vivo* intestinal permeation studies at optimal conditions of 25°C and 50% RH with a 12 hour light/dark cycles with continuous access to reverse osmosis (RO) water and pellets rat food (VRK Nutritional Solutions, Sangli, Maharashtra, India). The cages were labelled with the individual experiment title and the group names prior to the start of experiment to avoid error. The experiments were conducted in accordance to the Committee for the purpose of control and supervision of Experiments on Animals (CPCSEA) rules. ^58^

### *Ex vivo* intestinal permeation studies

The rats were randomly assigned to two groups *i.e* Group I: Plain CAR (n=3) and Group II: CAR-SCS (F8) (n=3). The number of animals per group was decided according to the existing literature. ^28^ These rats were euthanized ethically by giving an intraperitoneal injection of thiopental sodium overdose (50 mg/kg), ^23^^,^^30^ and the abdominal area was shaved and an incision of 5 cm was done. The intestine was removed and the ileocaecal junction was identified. This segment was then cleaned thoroughly by using the blunt end of the syringe and transferred to the Petri plate with Kreb’s Ringer buffer solution pH 7.4. This intestinal segment was then cut into pieces. One end was tightly tied with the thread. From the other side, 1 mL of the CAR, equivalent to 6.25 mg dispersed in the Kreb’s Ringer buffer solution pH 7.4 was added. Another end of the intestinal segment was sealed. Similarly, 1 mL of the CAR-SCS (F8), equivalent to 6.25 mg dispersed in the Kreb’s Ringer buffer solution pH 7.4, was added to another intestinal segment, followed by sealing of the intestine. The non-everted sacs were kept in 50 mL of Kreb’s Ringer buffer solution pH 7.4 in a magnetic stirrer with a 50 rpm speed aerated with oxygen utilizing a laboratory aerator (Atlas Air Pump 6000, Mumbai, Maharashtra). The samples were taken from the serosal compartment, *i.e.*, outside the sac, at 20, 40, 60, 80, 120, and 180 min, and a similar quantity of the fresh medium was incorporated. The samples were analyzed by UV-spectroscopy after filtration using 0.45 micron filters. The permeability of CAR from the CAR-SCS (F8) was obtained from the plot of the cumulative amount of CAR permeated from the rat intestine *vs.* time in min. The apparent permeability coefficient calculation was done as per the Equation 1 below. ^31^^,^^32^

$$P_{\mathrm{app}}=\frac{F}{A\times C_{0}}$$ (1)

Where A refers to the cross-sectional area of the intestinal segment (cm ^2^), F refers to permeation flux, C _0_ is the initial CAR concentration in μg/ml

### *In vivo* pharmacokinetic study

The rats were classified into three groups with each group containing 4 animals ((n=4 per group; total number of groups: 3).

Group A: Plain CAR (40 mg/kg)

Group B: CAR-Mannitol PM (powder equivalent to 40 mg/kg of CAR)

Group C: CAR-SCS (F8) formulation (powder equivalent to 40 mg/kg of CAR).

All the above samples were administered orally in the form of 0.2% carboxy methyl cellulose suspension. At the time intervals of 0.5, 1, 2, 4, 6, 12 and 24 hours, 200 μL of blood was collected from the retroorbital plexus in the centrifuge tubes with 10% EDTA. 100 μL of plasma was collected by centrifugation at 10,000 rpm for 10 min. ^20^^,^^29^

Plasma samples were analyzed for CAR by using high performance liquid chromatography (HPLC). The HPLC system (SHIMADZU LC2010-CHT, Schimadzu Corporation, Kyoto, Japan) with the dual piston pump, autosampler and a UV-visible detector was utilized. The obtained chromatograms were analyzed using the postrun icon in the LC solutions software version 5.57 (provided with the equipment used). The mobile phase utilized was Acetonitrile and water adjusted to pH 3.0 with trifluoroacetic acid (45:55). The flow rate used was 1 ml/min at the UV detector wavelength of 241 nm. A Kromasil C18 Reverse Phase column was utilized for the analysis.

The plasma samples were processed by protein precipitation method. 300 μL of methanol (precipitation agent) was incorporated into the tubes and was centrifuged for 1 min utilizing vortex mixer. This was centrifuged at 10,000 rpm for 10 min and separation of the supernatant. The supernatant was injected in the HPLC system. ^20^^,^^32^ The calibration plot for CAR in the plasma was plotted in the concentration range of 25 to 5,000 ng/mL, and showed a R ^2^ value of 0.983, depicting its linearity with the equation y=0.0014x+0.0897, where ‘x’ is the CAR conc. and ‘y’ is the peak area ratio of CAR to IS (Quetiapine).

### Stability studies

Optimized CAR-SCS (F8) was incorporated into the hard gelatin capsules, and the capsules were stored in the glass bottle at 40°C/75% RH for three months in a stability chamber (Thermolab Scientific Equipments, 500 L capacity). The drug content and DSC analysis for the samples was done at intervals of one, two, and three months.

## Results and discussion

### Preliminary trials

Initial trials were taken using two hydrophilic carriers *i.e.* mannitol and sorbitol for the preparation of CAR-SCS. For preliminary trials, CAR:hydrophilic carrier at a 50:50 ratio was extruded through the TSP. The kneading zone (B3) temperatures of 120-140°C were selected for the preparation of CAR-mannitol SCS and 70-110°C was used for CAR-sorbitol SCS based on the melting point of the hydrophilic carriers. Initially, the effect of different kneading zone temperatures on the SCS was assessed. For CAR-mannitol SCS, with the increase in kneading zone temperature to 140°C the product formed was sticky/cohesive in appearance, and the yield was very less owing to the loss of the product by sticking to the barrel. The increase in solubility was seen with the increase in kneading zone temperatures from 120-130°C beyond which the solubility decreased for CAR-mannitol SCS and the solubility increased with the increase in kneading zone temperature from 70-110°C for CAR-sorbitol SCS.

The impact of screw speed on the CAR-SCS formation was also examined. Screw speeds of 50-150 rpm were utilized. With an increase in the screw rpm to 150 rpm, the product yield was found to be more due to the decreased residence time in the TSP barrel resulting in the extrusion of fine powder. Additionally, the solubility of the product also enhanced which might be because of the increased speed resulting in the higher mixing action and shear created by the co-rotating screw, increased surface area and improved interaction of the water-soluble polymer with the drug at higher temperature. The solubility was found to be significantly enhanced with mannitol compared to sorbitol. Hence mannitol was screened as the hydrophilic carrier for preparing the CAR-SCS. From the initial trials ( Table 1), screw speed and the kneading zone temperature were found to have an effect on the solubility.

### Experimental design

Based on the preliminary trials, screw speed and kneading zone temperature were considered as important parameters having an impact on the CAR-SCS solubility. However, to assess the impact of the varying concentrations of mannitol and CAR, CAR:mannitol ratio was considered as a formulation related parameter for the statistical optimization by employing Design Expert software. BBD was employed to examine the influence of the three factors *i.e.*, CAR:mannitol ratio, kneading zone (B3) temperature, and screw rpm, on the solubility in 15 runs. The CAR: mannitol ratios selected were 20:80, 35:65, and 50:50. The kneading zone temperature and screw rpm ranged from 120-140°C and 50-150 rpm, respectively. The response variable was the solubility of CAR. For generating the statistical experimental design, Design Expert v.9.0.3.1 software was used. Different trials generated by Design Expert Software (F1 to F15), using the factors CAR:mannitol ratio, kneading zone temperature, and screw rpm, and the corresponding response, solubility is depicted in Table 2.

### Statistical analysis and optimization using BBD

**Figure 1.**
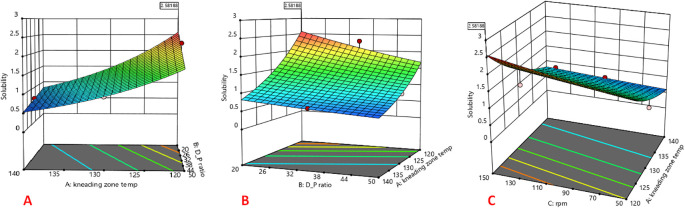
Response surface plot indicating the influence of (A) kneading zone temperature, (B) Drug: carrier ratio, and (C) screw rpm on solubility.

The 3D response surface plots are shown in Figure 1. The CAR:mannitol ratio, kneading zone temperature, and screw rpm were found to affect the solubility of CAR significantly. The response factor solubility was found to increase with decreasing kneading zone temperatures and increasing screw rpm. CAR-SCS showed maximum solubility at 120°C beyond which the solubility was found to decrease. It could be that, at the temperatures higher than 120°C, there could be some alterations in the interactions between the CAR and mannitol or changes in the physical properties of the materials or other factors like changes in the thermal characteristics, or viscosity which might have resulted in decreased solubility that need further investigation. Increase in the solubility with increase in screw rpm from 50 to 150 rpm might be because of the higher shear imparted to the mixture by the rotating screw. Solubility was affected by the CAR:mannitol ratio and showed a decrease with the increase in CAR:mannitol ratio. This could be because of the fact that, the increase in the CAR:mannitol ratio results in the decrease in the amount of hydrophilic mannitol available to bind with the increased amount of hydrophobic CAR. Hence, there is insufficient coverage of the hydrophobic CAR particles by the water-soluble carrier mannitol, causing a decrease in wettability and solubility of the product ANOVA analysis was done to estimate the effect of individual factors on the response. For the response, individual factors and model sum of squares was computed for linear, 2-factor interaction, quadratic and cubic models. The ANOVA results depicted that the model is significant with a F value of 34.47 and a p-value of <0.0001. The p-value less than 0.050 for the model indicates that the model is significant. Herein, CAR:mannitol ratio, kneading zone temperature and screw rpm are significant model terms. p-values for the model terms were <0.100 ( *i.e.* 0.040 for screw rpm, <0.0001 for kneading zone temperature and 0.0055 for CAR:mannitol ratio) indicating that the individual model terms are significant. A linear model was selected. The *R ^2^* value for the selected model was found to be 0.904. The coded equation in terms of the actual factors generated by DoE was as follows:

$$\ln\left(\text{solubility}\right)=-0.0290-0.0921\times \left(C:M\right)-0.248\times \mathrm{KZT}+0.0634\times \mathrm{rpm}$$

Where C:M represents CAR:mannitol ratio, KZT represents kneading zone temperature and rpm represents screw rpm.

The validation of the model was done by comparing the practical values with the values generated by the design expert software and calculating % residual by using the Equation 2:

$$\%\text{residual}=\frac{\text{Predicted results}-\text{Observed results}}{\text{Predicted results}}\times 100$$ (2)

The optimized trial suggested by the Design Expert Software was utilized for validation. The trial was conducted using the 20:80 ratio of Drug:mannitol at a kneading zone temperature of 120°C showed a saturation solubility of 2.45. The solubility predicted by the software was 2.1 mg/mL. This accounted for the % residual of ±14.3%. The DoE software showed the desirability for the optimized factors as 1.00.

### Molecular dynamic (MD) simulation studies at different kneading zone temperatures, stability determination, and trajectory analysis

Molecular Dynamic (MD) studies were performed for the CAR-SCS to interprete the interaction of CAR with mannitol at different ratios employed in the current work i.e 20:80, 35:65, and 50:50 and different kneading zone temperatures (120°C, 130°C and 140°C) to determine the best ratios which would improve the solubility of CAR. In the starting phase of MD simulation, the CAR-SCS was stabilized. According to the hydrophilic and hydrophobic report generated for all CAR-SCS from the *in-silico* MD studies, as depicted in the Table 3, the CAR-SCS with a 20:80 ratio of CAR: mannitol, prepared at the kneading zone temperatures of 120°C and those prepared with 35:65 ratio of CAR:mannitol with the kneading zone temperatures of 120°C, 130°C, 140°C were predicted to be the optimal ones which could enhance the CAR solubility. These CAR-SCS were found to show more hydrophilicity and less hydrophobicity compared to the other CAR-SCS as per the *in-silico* hydrophilic and hydrophobic report.

**Table 3.** Hydrophilic and hydrophobic report form Molecular Dynamics simulation studies.

Ratios of drug:mannitol	Kneading zone temperature (°C)	Hydrophilic portion	Hydrophobic portion
20:80	120	2582.11	688.86
20:80	130	2140.41	529.47
20:80	140	2496.22	676.12
35:65	120	2804.52	819.49
35:65	130	2833.62	999.50
35:65	140	2914.20	734.13
50:50	120	2155.27	1032.32
50:50	130	2255.051	893.723
50:50	140	2622.993	1123.653

**Figure 2.**
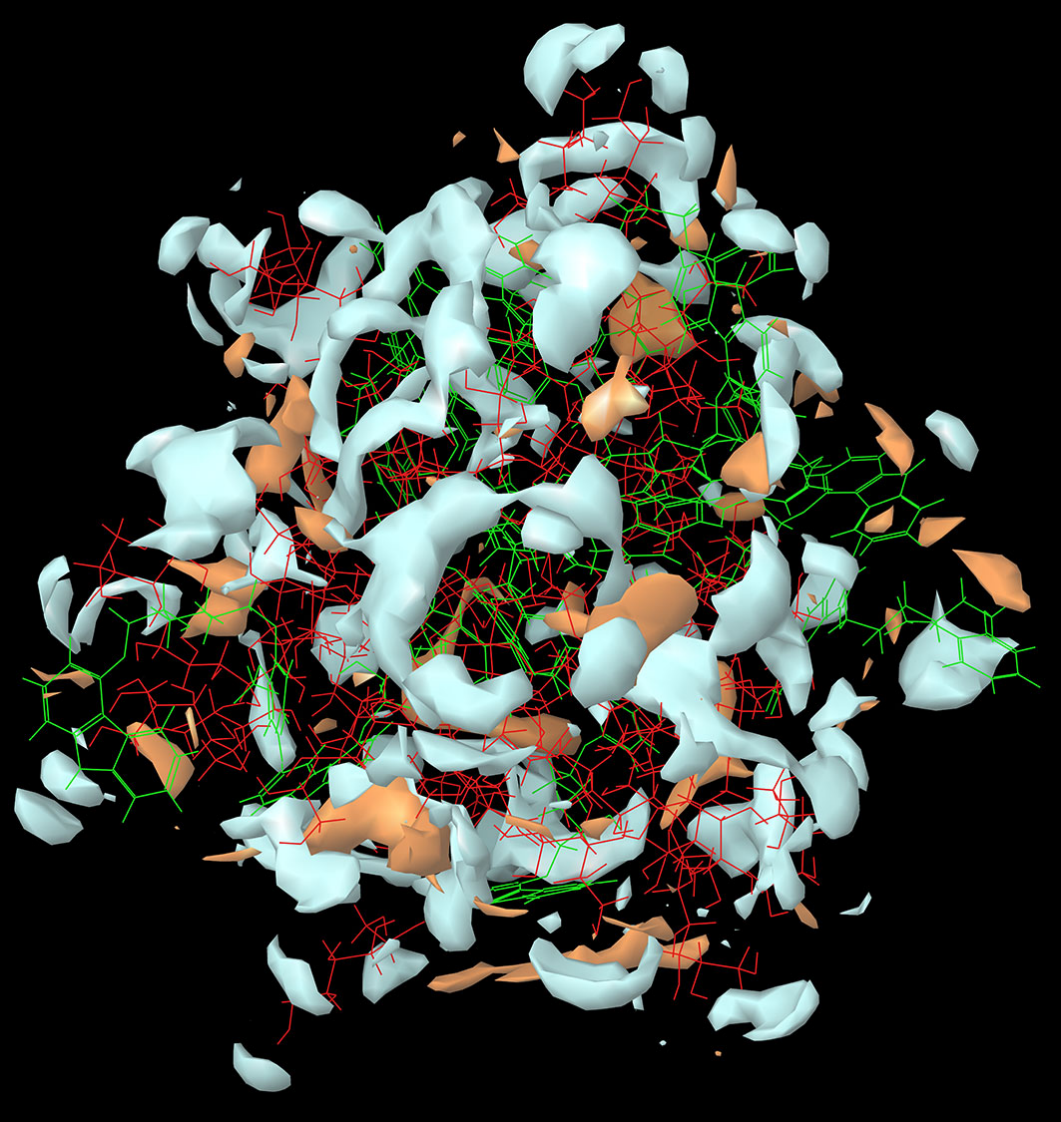
The hydrophilicity and hydrophobicity of the Carvedilol-solid crystal suspension prepared using 20:80 ratio of carvedilol: mannitol at the kneading zone temperature of 120 °C. (Hydrophilic portion is represented in white color and the orange color depicts the hydrophobic portion).

**Figure 3.**
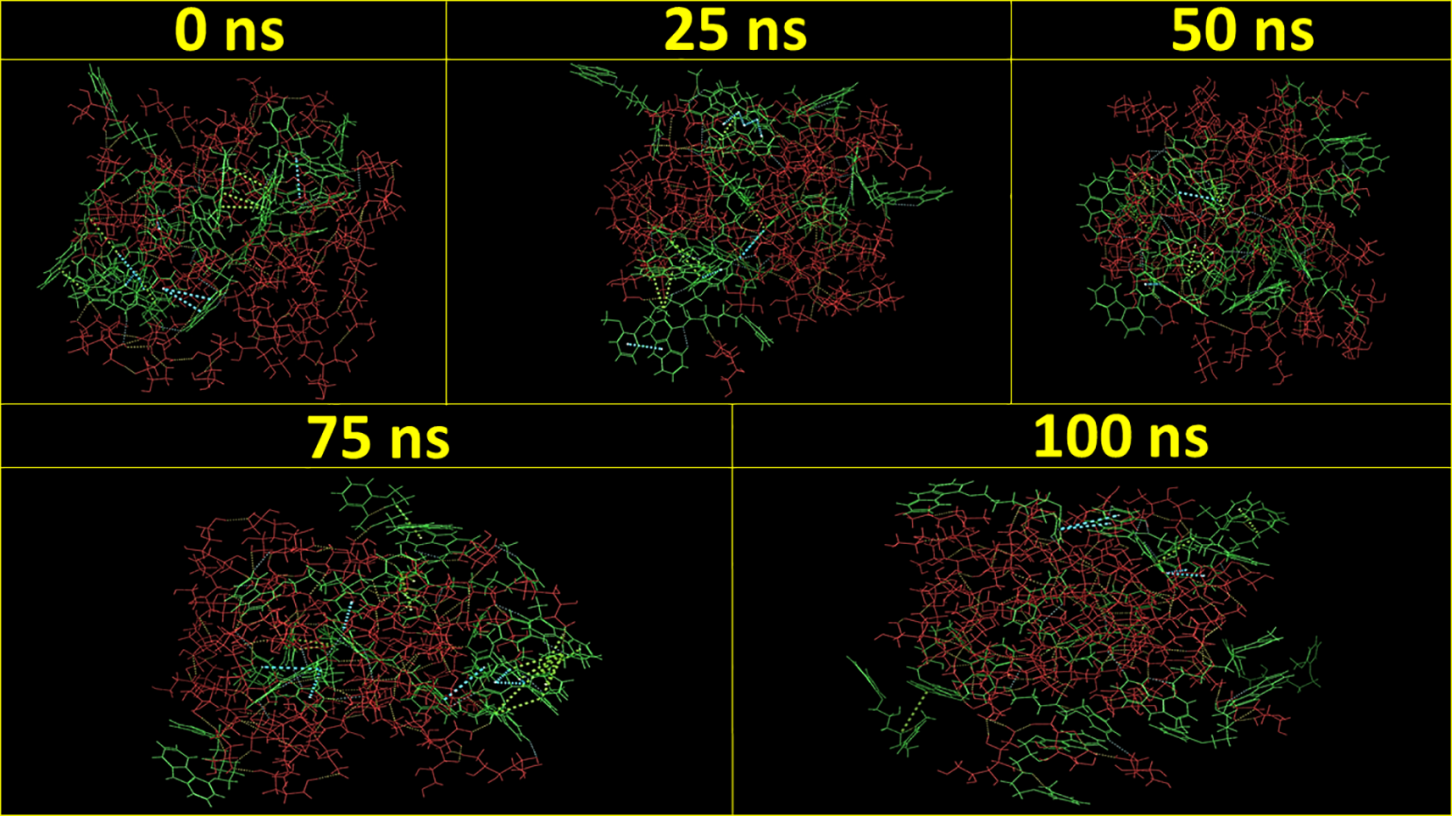
Molecular Dynamic simulation for the Carvedilol-solid crystal suspension prepared using 20:80 ratio of Carvedilol:mannitol at the kneading zone temperatures of 120 °C for 0 ns, 25 ns, 50 ns, 75 ns, and 100 ns.

**Figure 4.**
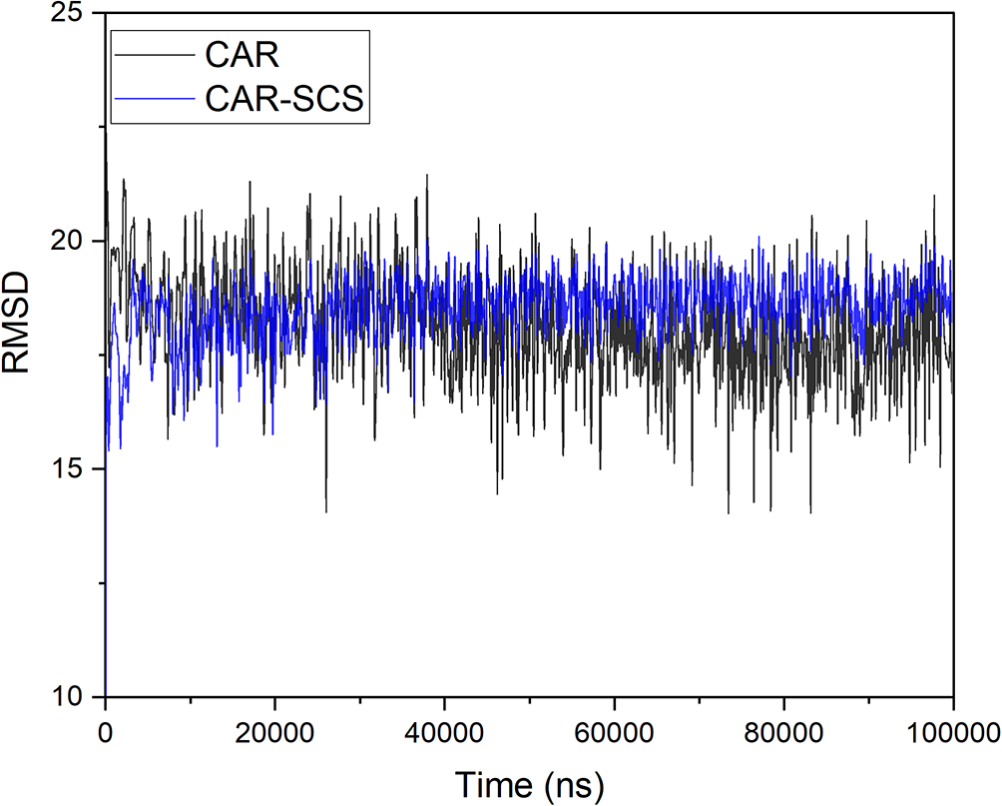
Root mean square deviation (RMSD) plotted in between the original carvedilol structure and the structures computed during molecular dynamic simulation of Carvedilol-Solid crystal suspension prepared using 20:80 ratio of carvedilol:mannitol at the kneading zone temperature of 120 °C.

The results of the *in-silico* molecular simulation studies were similar to the experimental DoE optimized result. As per the experimental results, the CAR-SCS formulation prepared using a 20:80 ratio of CAR:mannitol at the kneading zone temperatures of 120°C showed the best results. The hydrophilicity and hydrophobicity generated from the *in-silico* MD studies of the CAR-SCS (F8) prepared using 20:80 ratio of CAR:mannitol at the kneading zone temperatures of 120°C is depicted in Figure 2. As illustrated by the molecular simulation studies, hydrophilic portion is represented in white color and the orange color depicts the hydrophobic portion. The hydrophilicity and hydrophobicity of all the CAR-SCS formulations predicted by *in silico* MD studies are illustrated in Table 3. For assessing the stability of the CAR-SCS structures during the MD simulation, the structures from the trajectory were aligned with mannitol atoms, and the root mean square deviation (RMSD) calculation was done separately for CAR and CAR-SCS at different temperatures and ratios with respect to the preliminary frame. The fluctuation in the RMSD values was found to be less than 5Å. This indicated a very low fluctuation showing the stability of CAR-SCS complex. Figure 3 demonstrates the MD simulation done for the CAR-SCS formulation prepared using a 20:80 ratio of CAR: mannitol at the kneading zone temperatures of 120°C. The RMSD graphs were plotted for the original CAR structure and the MD simulation computed structures of optimum CAR-SCS. The RMSD plots for optimum CAR-SCS prepared using a 20:80 ratio of CAR:mannitol at the kneading zone temperatures of 120°C are indicated in Figure 4. The MD simulation studies showed the stability of the CAR-SCS with the least fluctuation in the RMSD values.

### Characterization

***Differential scanning calorimetry (DSC)***

**Figure 5.**
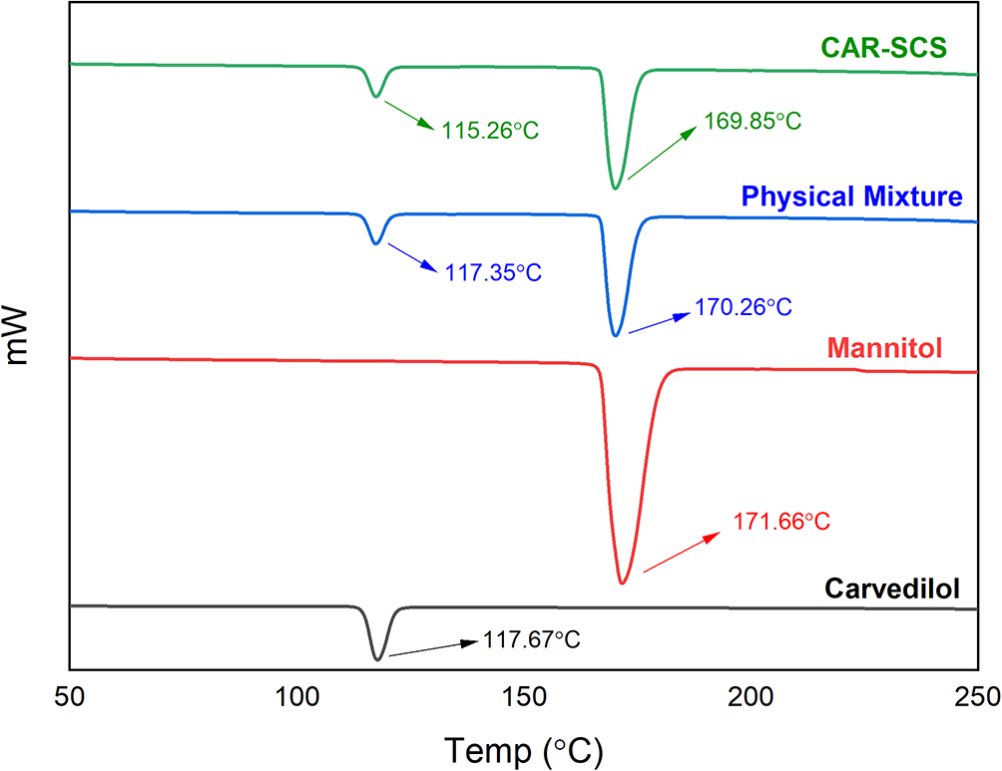
Differential Scanning Calorimetry (DSC) spectra of (i) Carvedilol (ii) Carvedilol-mannitol Physical mixture and (iii) Carvedilol -Solid crystal suspension (F8) samples. Physical mixture-Carvedilol: mannitol physical mixture, CAR-SCS-Optimized Carvedilol-solid crystal suspension (F8).

DSC thermograms of CAR, mannitol, PM, and the CAR-SCS (F8) are depicted in Figure 5. A sharp endotherm at 117.67 °C was observed for CAR revealing the crystallinity of the drug. Mannitol exhibited a characteristic endotherm at 171.66°C, confirming the crystallinity of mannitol. Two melting endotherms were seen in the DSC spectra of the PM and the CAR-SCS (F8). In CAR-SCS (F8), the melting endotherm of mannitol and the CAR were less intense and was slightly shifted to the low temperature compared to the plain mannitol. This was attributed to the presence of the molten form of mannitol reducing the melting point of higher melting point substances. ^4^^,^^5^ The DSC results for the CAR-SCS (F8) confirm the crystallinity of the CAR-SCS (F8) and indicate that both CAR and mannitol occur as distinct crystalline phases in CAR-SCS (F8). The least melting point depression shows that mannitol creates a very poor solvating environment for CAR but is effective in aiding the efficient wetting of the CAR. ^4^

***Thermogravimetry analysis (TGA)***

**Figure 6.**
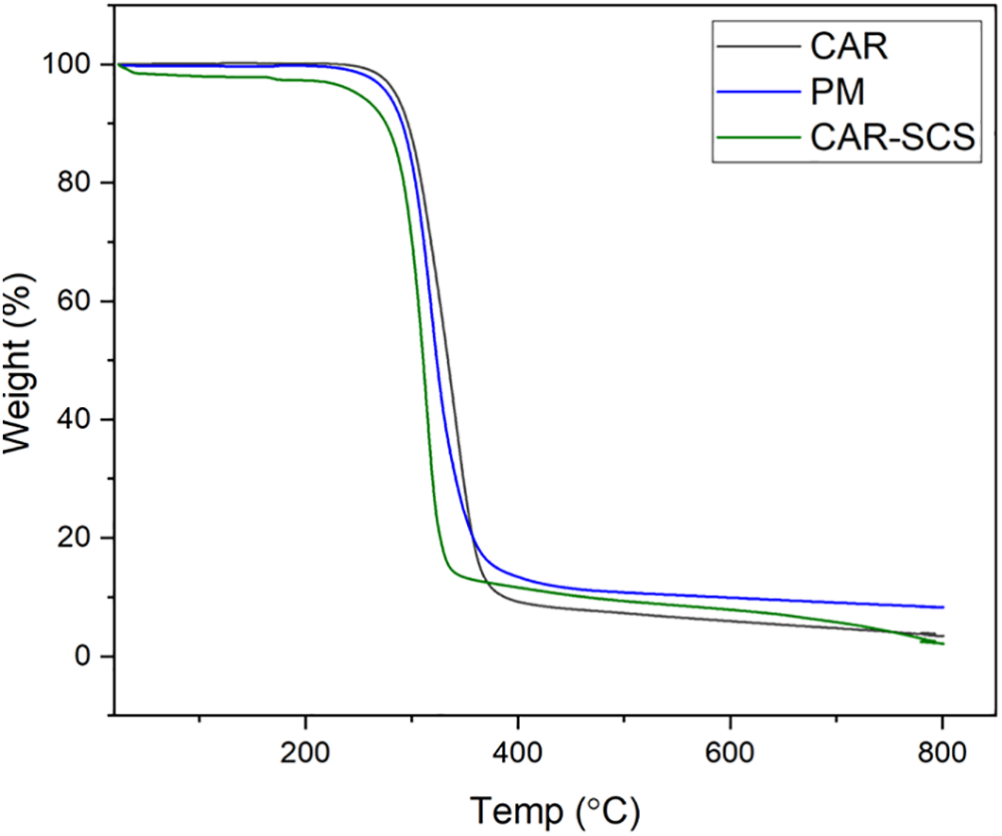
Thermogravimetry analysis thermograms of (i) Carvedilol (ii) Carvedilol -mannitol Physical mixture and (iii) Carvedilol -Solid crystal suspension (F8).

TGA was performed to assess the thermal stability of CAR, PM and CAR-SCS (F8). The TGA curves of CAR, PM and the CAR-SCS (F8) are shown in Figure 6 which shows that a slight loss in the weight by 5% w/w for all the samples was observed at 245-275°C which is due to the presence of absorbed water and water of crystallization. The TGA curve revealed that the drug is thermally stable up to 222°C which is similar to the previously reported value. ^35^ The decomposition range of carvedilol was from 300-800°C. The decomposition of PM containing the CAR and mannitol started at 219°C and for CAR-SCS (F8) formulation, the decomposition started at 245°C. Hence, the thermal stability of CAR-SCS (F8) formulation was similar to that of the plain CAR and the PM indicating no change in the thermal properties of CAR after forming CAR-SCS using a TSP. At the temperatures above 300°C, the samples showed 70% of weight loss.

### Fourier transform infrared (FTIR) spectroscopy

**Figure 7.**
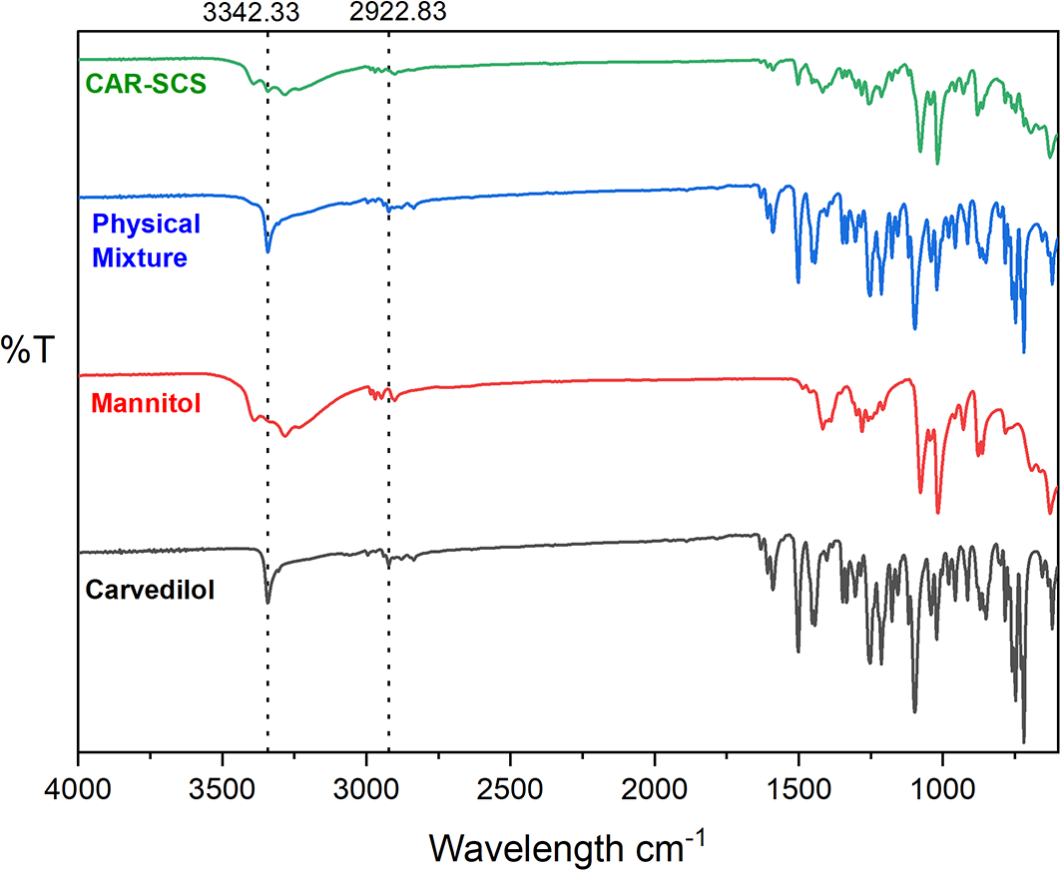
Fourier Transform Infrared (FTIR) spectra of different samples: Carvedilol, mannitol, Physical mixture and Carvedilol-Solid crystal suspension (F8). Physical mixture-Carvedilol: mannitol physical mixture, CAR-SCS: Optimized Carvedilol-solid crystal suspension (F8).

The overlay FTIR spectra of CAR, mannitol, PM, and CAR-SCS (F8) is illustrated in Figure 7. FTIR spectrum of CAR exhibited typical peaks as reported previously in the literature ^36^: a peak at 3342.33 cm ^-1^ depicting the –N-H stretch; the C-H stretch in the region is 2922.83 cm ^-1^; the peaks from 1251.69 cm ^-1^ to 1453.40 cm ^-1^ relating to the C-C stretch were observed. The band from 1453.4 cm ^-1^ to 1501.8 cm ^-1^ was allocated to C=C stretch. The FTIR spectra of mannitol exhibited the characteristic peaks as reported earlier in literature ^37^ with a peak at 2948.06 cm ^-1^ depicting the –C-H group, and at 1077.66 cm ^-1^ depicting the C-O stretch. CAR-SCS (F8) showed the presence of peaks characteristic to both CAR and mannitol. The sharp characteristic peak of CAR at 3342.33 cm ^-1,^ which could be seen even in the 1:1 physical mixture of CAR and mannitol, was observed in the CAR-SCS (F8).

### Powder X-ray diffraction (XRD) studies

**Figure 8.**
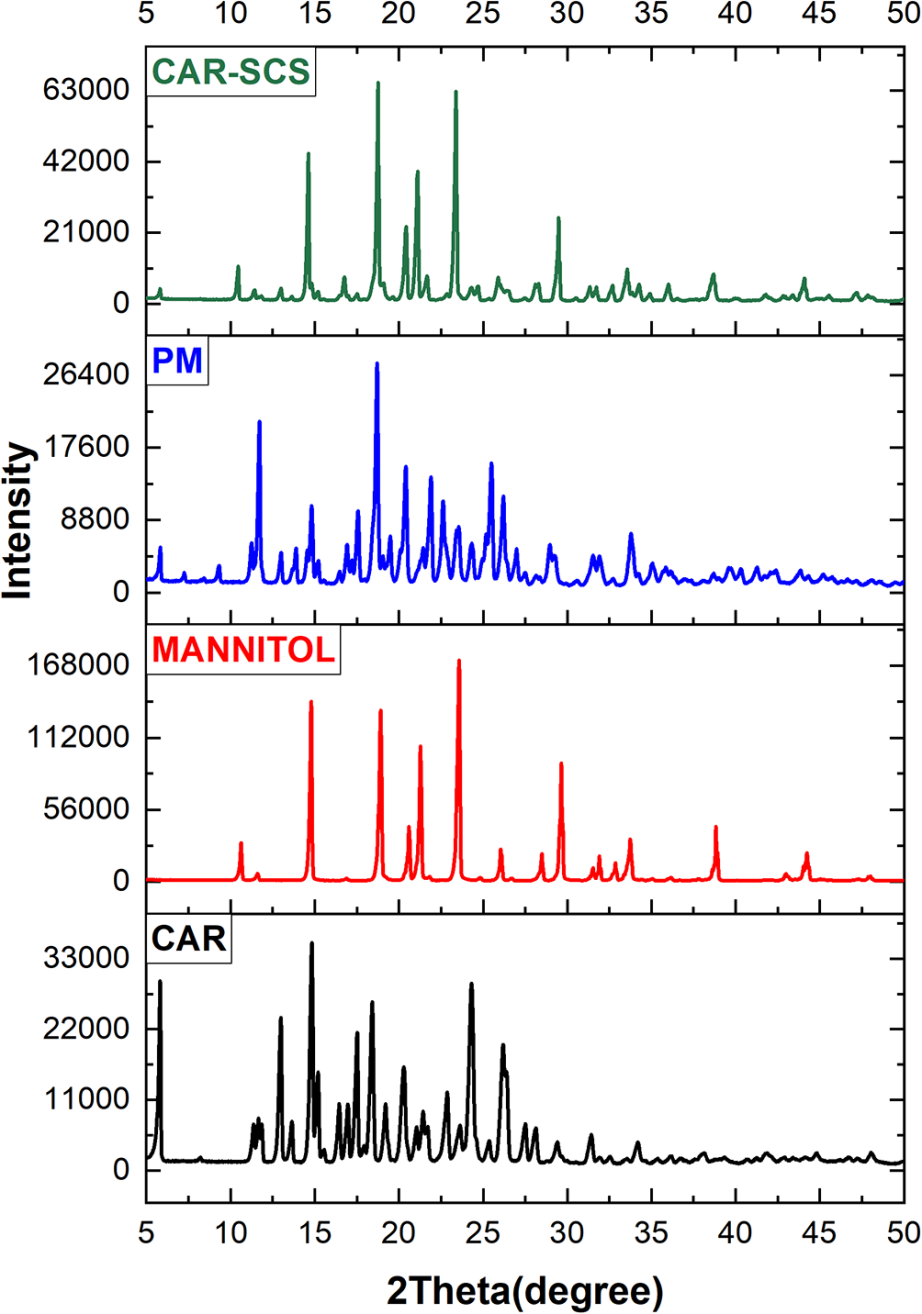
X-ray diffraction (XRD) pattern of: Carvedilol, mannitol, Physical mixture and Carvedilol-Solid crystal suspension (F8).

Powder XRD is a sensitive technique and has been considered a standard method for phase identification as the PXRD pattern is directly linked with the crystal structure of the materials. The significant change in the XRD patterns represents the change in the phase composition. ^38^ The XRD patterns of CAR, mannitol, physical mixture of CAR-mannitol, and CAR-SCS (F8) are depicted in Figure 8. The XRD patterns of CAR, mannitol, CAR-mannitol physical mixture, and CAR-SCS (F8) were found to be sharp, confirming their crystalline nature.

The XRD pattern of the CAR-SCS (F8) revealed the presence of characteristic crystalline peaks of both CAR and mannitol suggesting the combination of XRD patterns of CAR and Mannitol.

### Scanning electron microscopy (SEM) and energy dispersive spectroscopy (EDS)

**Figure 9.**
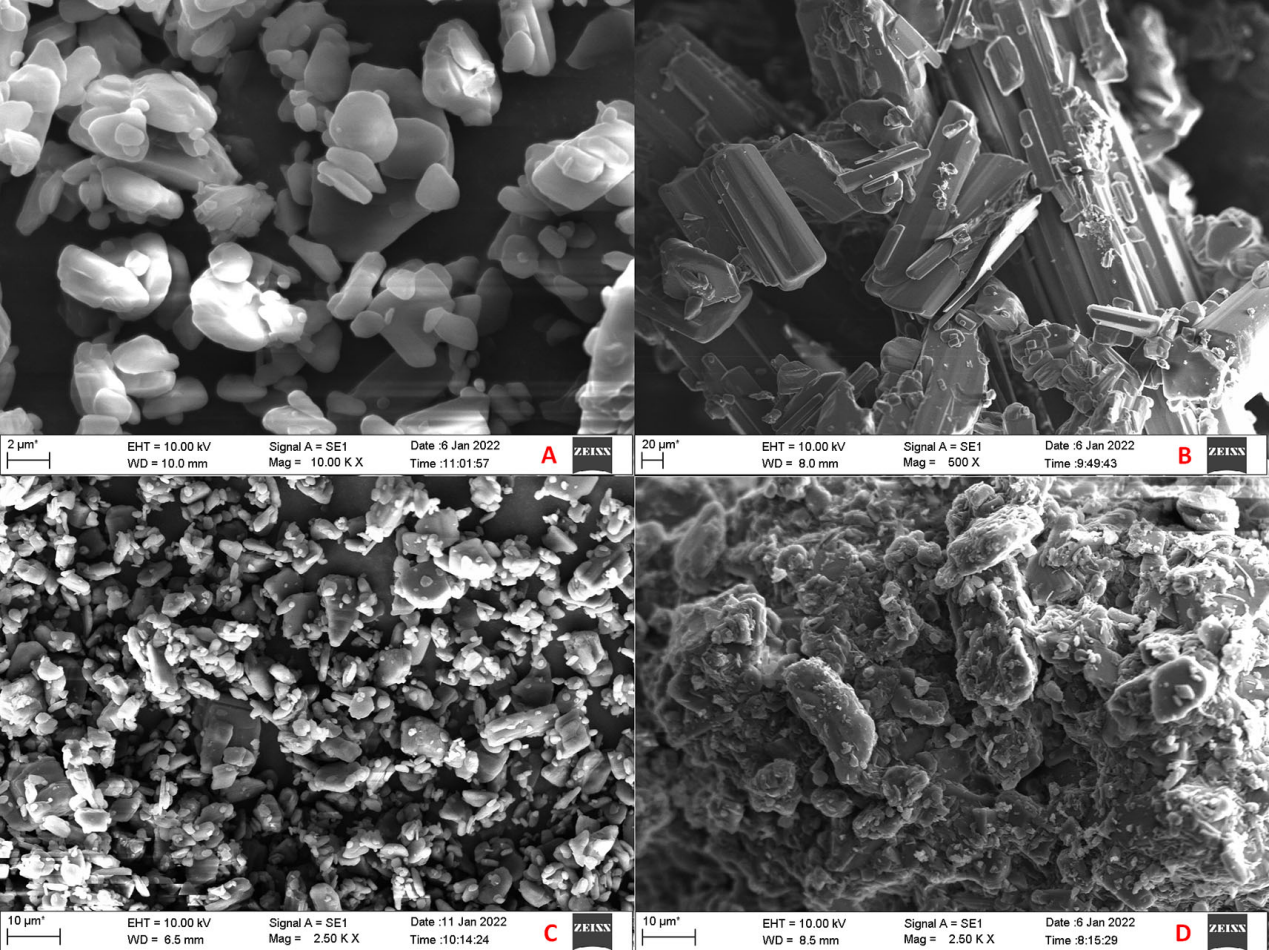
Scanning electron microscopy (SEM) images. A: Carvedilol; B: Mannitol; C: Carvedilol:Mannitol Physical Mixture; D: Carvedilol-Solid crystal suspension (F8).

**Figure 10.**
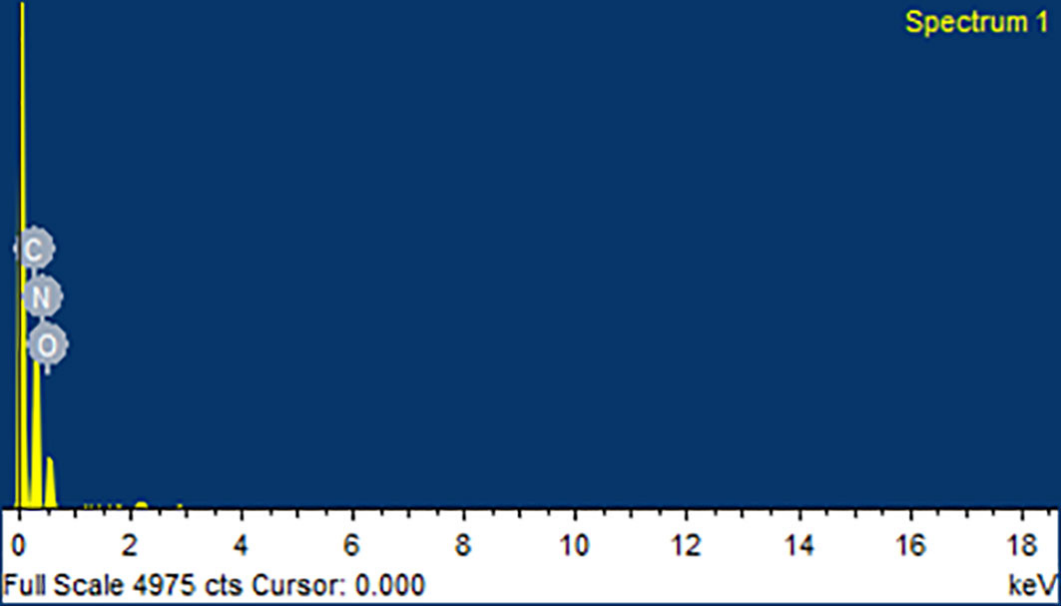
Energy dispersive spectroscopy (EDS) for the optimized Carvedilol-Solid crystal suspension (F8) formulation.

The SEM images of CAR, mannitol, PM and CAR-SCS (F8) are presented in Figure 9. From the SEM analysis, it was observed that CAR showed an irregular shape with a very smooth surface. The appearance for pure CAR was similar to the priorly reported data. ^40^ Mannitol showed needle-like morphology. The physical mixture was more of a mixture of CAR with the hydrophilic carrier mannitol. The SEM image of the CAR-SCS (F8) formulation revealed consistent dispersion of the CAR in the melted carrier matrix. As the appearance of the CAR-SCS (F8) was significantly different from that of the starting materials (i.e. CAR and mannitol), it signified an interaction between CAR and mannitol to form a homogenous CAR-SCS (F8) formulation. EDS technique was used to study the elemental composition of the CAR-SCS (F8). The data obtained from EDS is illustrated in Table 4. The EDS data for the CAR-SCS (F8) formulation displayed the presence of carbon, nitrogen, and oxygen in the chemical structure of the CAR-SCS (F8), as can be observed in Figure 10. Since, mannitol does not contain nitrogen, the presence of nitrogen in the EDS results for CAR-SCS (F8) formulation was concluded to have come from the CAR.

**Table 4.** Energy dispersive spectroscopy (EDS Data for Carvedilol -Solid crystal suspension (F8).

Element	% weight	Atomic weight
Carbon (C)	58.54 ± 1.48	65.15
Nitrogen (N)	1.63 ± 2.07	1.55
Oxygen (O)	39.83 ± 1.18	33.28
Total	100.00	100.00

### Raman spectroscopy

**Figure 11.**
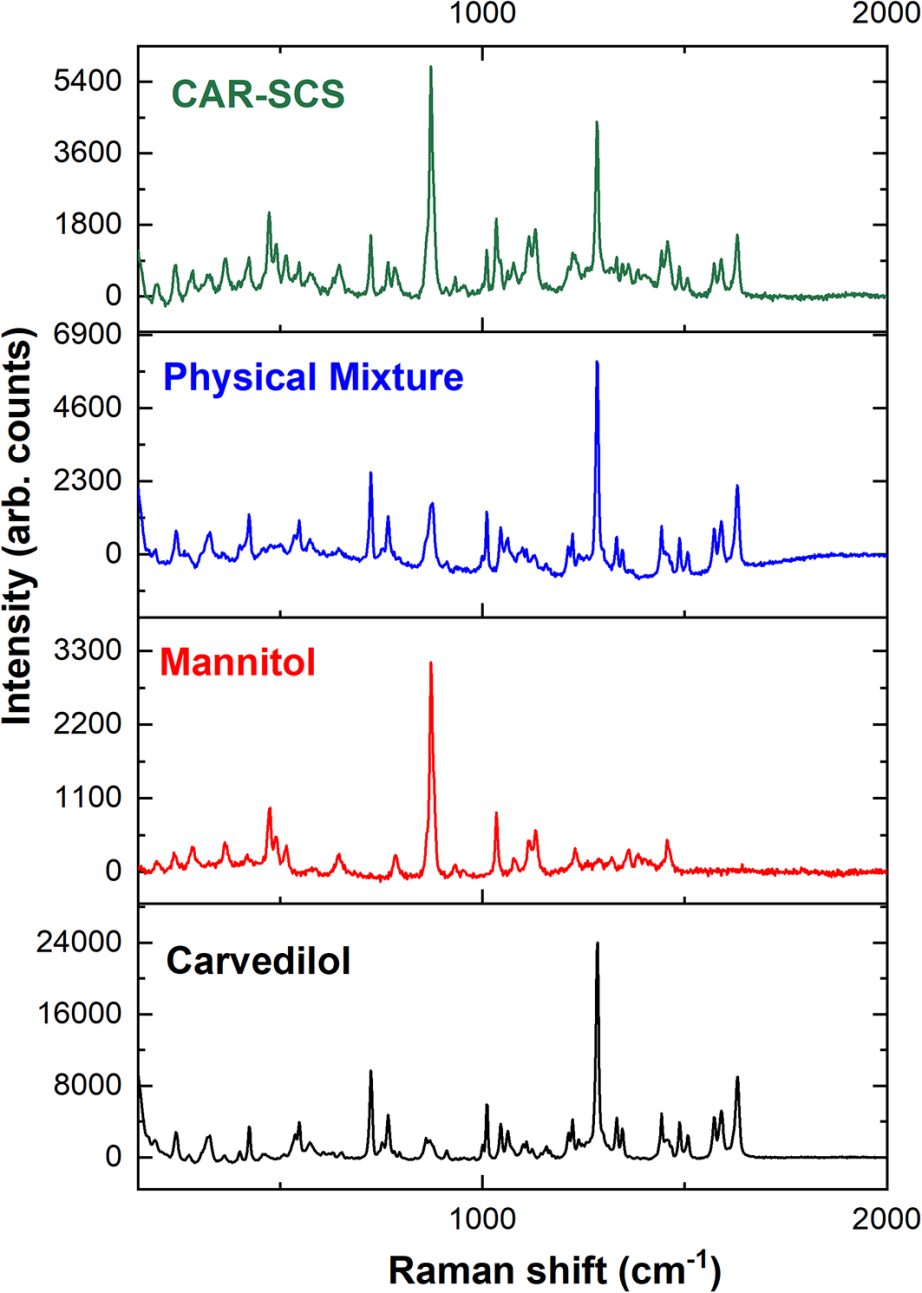
Raman Spectroscopic data of different samples: Carvedilol, mannitol, Physical mixture and Carvedilol-Solid crystal suspension (F8). Physical mixture-Carvedilol:mannitol physical mixture, CAR-SCS: Optimized Carvedilol-Solid crystal suspension (F8).

The Raman spectra of CAR, mannitol, physical mixture, and the CAR-SCS (F8) are illustrated in Figure 11. Raman analysis was done to understand the distribution of the CAR in the carrier, and to determine the crystallinity of the CAR in the optimized CAR-SCS (F8) formulation. Usually, CAR shows well-defined peaks in the Raman spectra owing to its crystallinity, whereas the broad spectra is observed if the drug is in the amorphous form. ^42^ The Raman spectra of CAR were found to show sharp peaks characteristic of CAR. ^43^^,^^44^ The carrier mannitol was also found to exhibit sharp peaks, as reported in the literature. ^44^ The optimized CAR-SCS (F8) were found to show sharp peaks similar to the CAR and mannitol, indicating the crystallinity of the starting materials and their mixture. The optimized CAR-SCS (F8) showed the distribution of crystalline CAR in the mannitol carrier, showing the presence of characteristic peaks present in CAR and mannitol.

### Nuclear magnetic resonance (NMR) analysis

**Figure 12.**
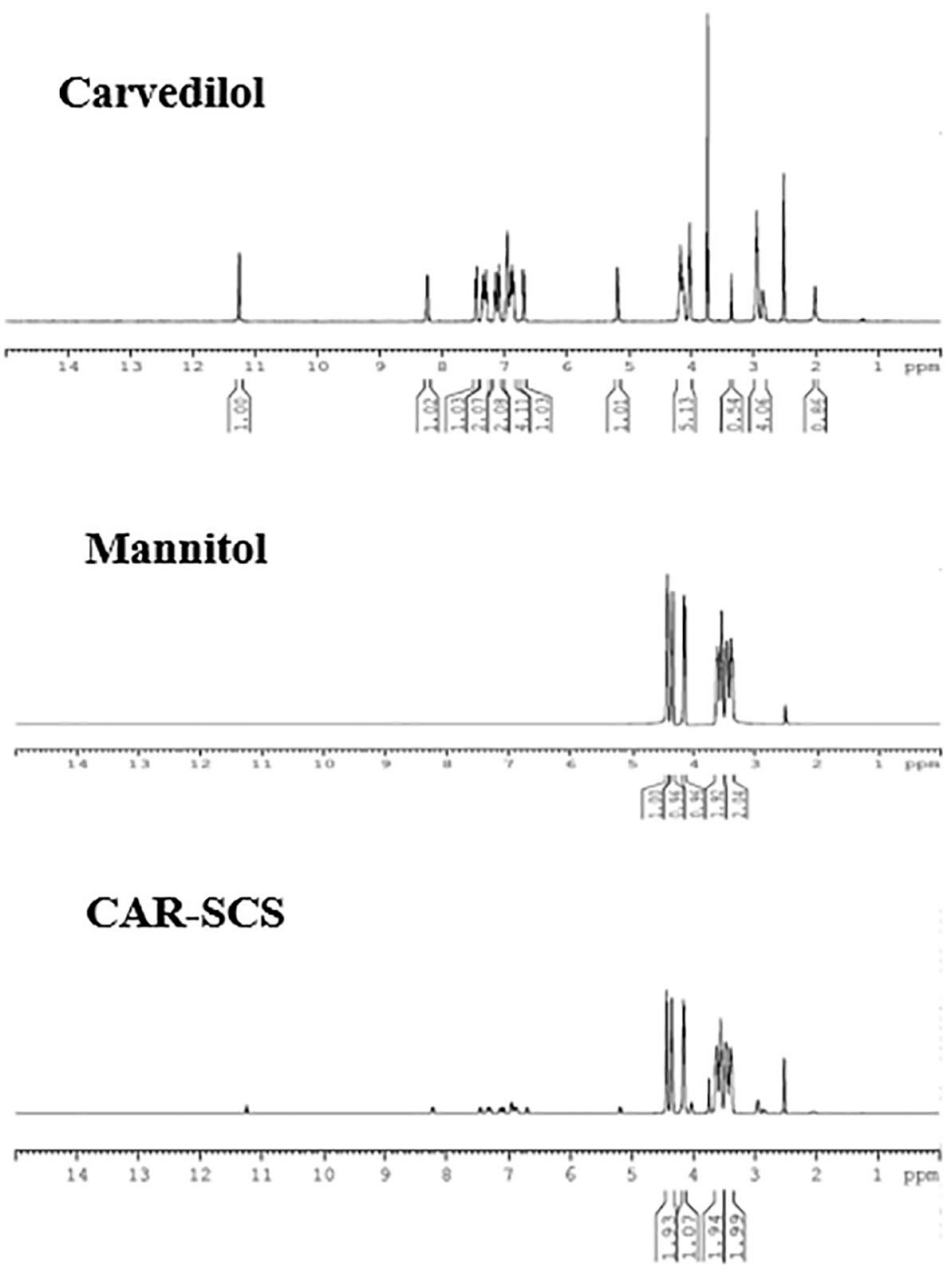
Nuclear magnetic resonance (NMR) spectra of Carvedilol, mannitol and optimized Carvedilol-Solid crystal suspension formulation CAR-SCS(F8).

CAR, mannitol and optimized CAR-SCS (F8) were characterized by NMR analysis ( Figure 12). A sharp singlet obtained for the sample, CAR at 3.75 ppm was assigned to the three protons of –CH _3_ group. The multiplets at 6.895-6.942 ppm and 6.680-6.852 ppm owing to the presence of four aromatic protons were seen. The multiplets at 2.859-2.946 ppm and 4.021-4.139 ppm were because of the two –CH _2_ protons. -NH amine proton displayed a singlet at 2 ppm. –NH indole proton showed a singlet at 11.26 ppm, and a –OH proton showed a doublet at 5.175-5.183 ppm. The four protons from the indole rings showed four doublets at 7.356-7.442, 6.680-6.700, 7.274-7.293, and 7.066-7.085 ppm. A doublet at 8.221-8.241 was assigned to the isolated proton of the indole ring. ^45^

The NMR spectra of mannitol showed the characteristic hydrogens bonded to the oxygen at 4.140, 4.342, and 4.427 ppm. The symmetrical hydrogens of mannitol were detected at 3.380 ppm, 3.462 ppm, 3.525 ppm, and 3.604 ppm. ^46^ The ^1^H NMR spectra of the CAR-SCS (F8) visibly depicted presence of proton signals of both CAR and mannitol, indicating the non-covalent interaction between CAR and mannitol, thus forming CAR-SCS (F8).

### Saturation solubility studies

**Figure 13.**
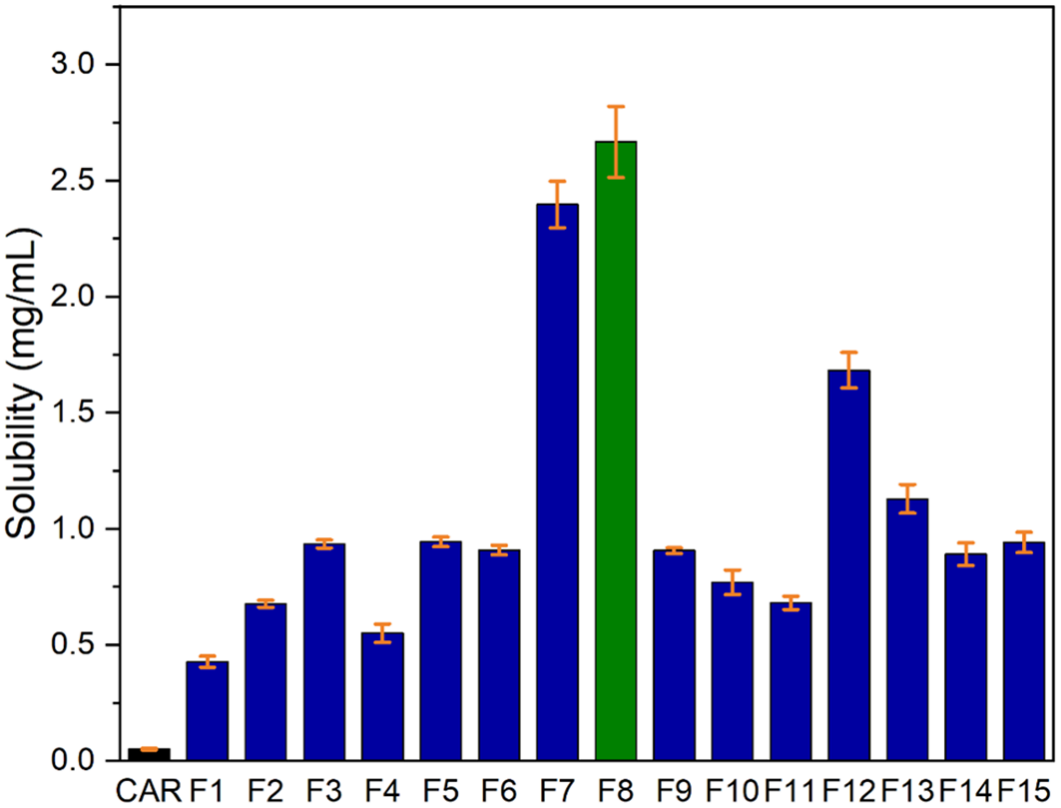
Saturation solubility of Plain Carvedilol and Carvedilol-solid Crystal suspension formulations.

As reported previously in literature, Carvedilol exhibits pH dependent solubility with good solubility with little increase in solubility of the pure drug as the pH increases from 1.2 to 4.6 and decreased after pH 6.8. ^47^ A buffer of pH 6.8 was selected to perform the saturation solubility studies of all the CAR-SCS batches. The solubility of CAR in pH 6.8 buffer after 48 hours was estimated to be 0.05 mg/mL, which was similar to the earlier reported value. ^49^ The CAR-SCS (F8) showed solubility of 2.5 mg/mL, as illustrated in Figure 13. CAR-SCS (F8) showed a 50-fold increase in solubility, probably because of the hydrophilic nature of mannitol facilitating wetting and hydration of the poorly soluble drug. Molten mannitol acts as a hydrophilic carrier for CAR in the twin screw processing process, thus intimately mixing with CAR and surrounding it, resulting in improved solubility by enhancing the wetting of drug particles and increasing the surface area compared to the plain CAR. The increase in the solubility seen for the CAR-SCS (F8) was much higher compared to the previously reported studies. ^3^^,^^49^

### Determination of flow properties of the optimized CAR-SCS (F8) formulation

The flow properties of the CAR-SCS (F8), including tap density, angle of repose, bulk density, and Hausner’s ratio, were evaluated. These parameters were compared with the pure CAR. Powders can possess good flow or could be sticky/cohesive. The critical knowledge regarding the powder flow properties is of utmost importance especially during manufacturing of pharmaceutical dosage forms. ^48^ The powder flow behaviour could impact the weight or the content uniformity of the pharmaceutical dosage form. Hence, determination of powder flow properties is important. The parameters for the plain CAR and the CAR-SCS (F8) are illustrated in the Table 5. The angle of repose for pure CAR showed a value of 47.6°, indicating poor flow. The compressibility index was 24.2% and Hausner’s ratio was 1.32 for the pure drug indicating passable flow. The CAR-SCS (F8) showed an excellent angle of repose of 25.73°, indicating considerably improved flow properties compared to the plain CAR. The compressibility index and Hausner’s ratio of CAR-SCS (F8) formulation were 20.2% and 1.25, respectively demonstrating fair flow properties. ^51^ These results indicated improved micromeritic properties in the CAR-SCS (F8) in comparison with pure CAR.

**Table 5.** Flow properties of Carvedilol and Carvedilol-solid Crystal suspension (F8) formulation.

Parameters	Pure CAR	Optimized CAR-SCS (F8)
Bulk density (g/mL)	0.25	0.16
Tapped density (g/mL)	0.33	0.19
Angle of repose	47.60	25.73
Compressibility index (%)	24.20%	20.20%
Hausner’s ratio	1.32	1.25

### Practical yield and drug content

The practical percent yield for all the CAR-SCS formulations was found to be between 20.40 to 91.4%. The content of the CAR in all the SCS formulations ranged from 51.20-99.49%. The % practical yield and % drug content of all the CAR-SCS is depicted in Table 6. Formulations F8 and F3 showed better % yield and % drug content, respectively. Better % yield obtained for the F3 could be because of the higher screw speed utilized for the preparation of the same. Better % drug content for F8 formulation could be because of the optimal temperatures and screw speed utilized for the F8 batch resulting in a good flow through the barrel with least drug sticking to the barrel and better % yield.

**Table 6.** Percentage practical yield and Percent drug content for all Carvedilol-solid crystal suspension formulations.

Formulation code	Percentage yield (%)	Percent drug content (%)
F1	21.20	74.20
F2	48.00	65.55
F3	72.90	99.49
F4	41.00	63.99
F5	32.20	96.70
F6	26.60	83.20
F7	22.20	56.10
F8	91.60	77.75
F9	20.40	55.72
F10	46.10	73.14
F11	28.10	60.00
F12	64.90	51.20
F13	43.50	85.64
F14	33.80	64.57
F15	14.60	54.97

### *In vitro* dissolution study

**Figure 14.**
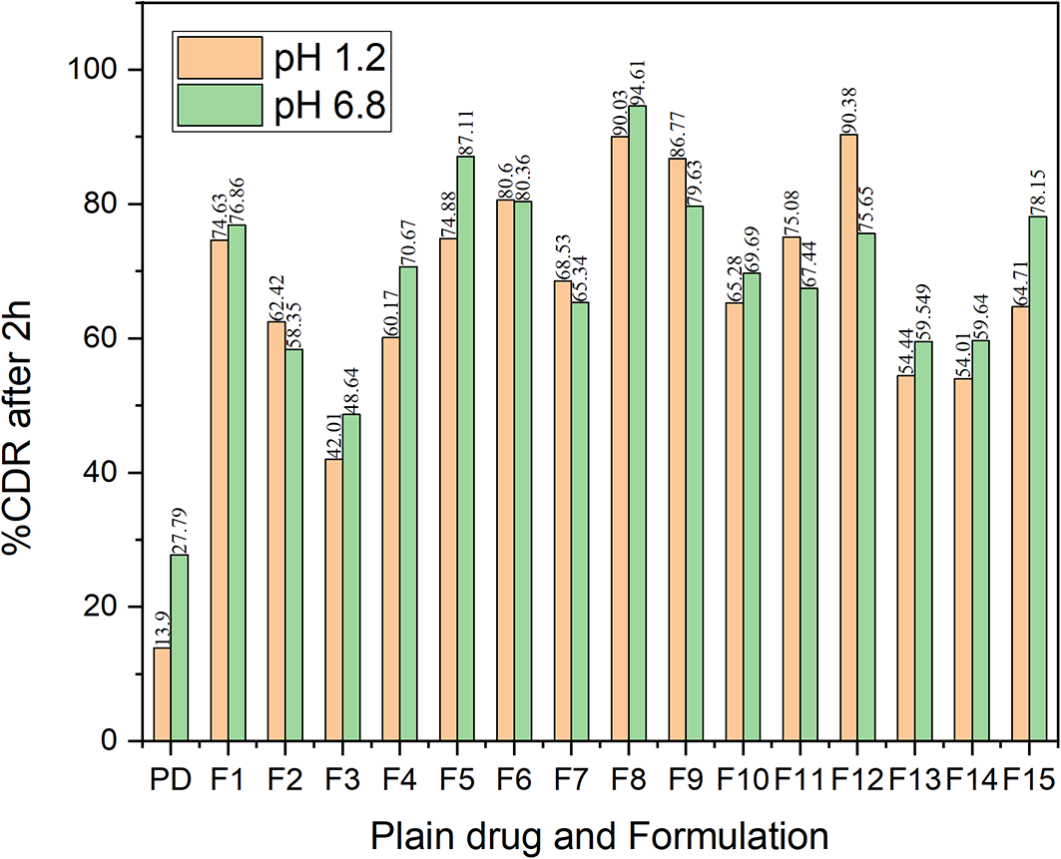
% Cumulative drug release of Carvedilol in its plain form and in different CAR-SCS formulations at pH 1.2 and pH 6.8 in dissolution studies. PD: Plain Carvedilol.

**Figure 15.**
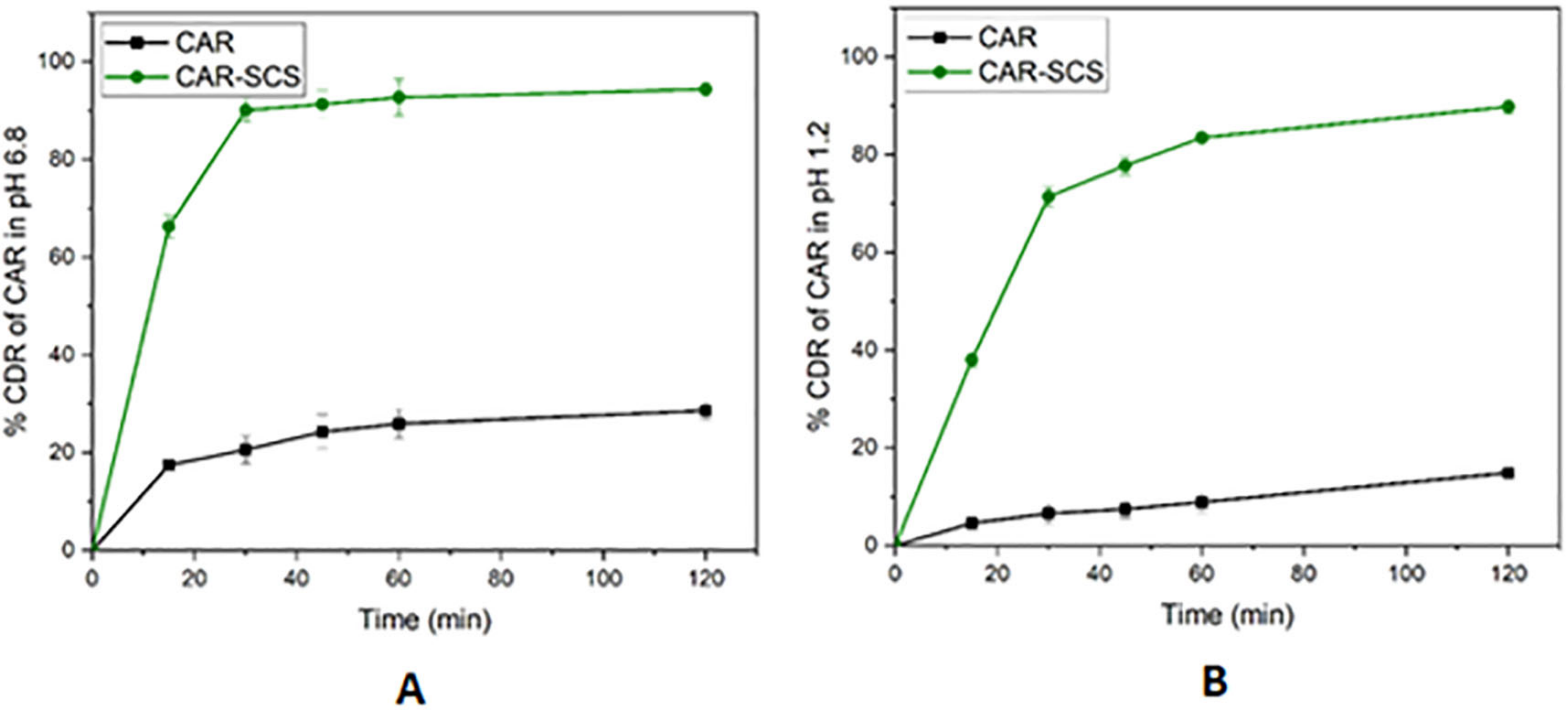
Comparative dissolution profiles of Carvedilol (plain CAR) and F8 formulation (optimized Carvedilol-Solid crystal suspension formulation) in different dissolution media. (A) pH 6.8 buffer and (B) HCl solution (pH 1.2).

The dissolution study was conducted in pH 1.2 of HCl solution as well as phosphate buffer solution of pH 6.8. Sodium lauryl sulfate (SLS) was incorporated in the dissolution medium as the addition of surfactant helps to accelerate the dissolution rate by acting as solubilizing agent. ^49^ The dissolution characteristics of all the formulations indicated a better dissolution rate than the bulk drug in both pH 1.2 and pH 6.8 buffer ( Figure 14). The comparative dissolution profiles of the drug and the CAR-SCS (F8) formulation in pH 1.2 HCl solution and pH 6.8 buffers are represented in Figure 15 (A) and (B). The *in vitro* dissolution studies for the CAR-SCS (F8) revealed a 6.03- and 3.40-times enhancement in dissolution rate as compared to the plain CAR in pH 1.2 HCl solution and pH 6.8 phosphate buffer respectively. Release of pure CAR was found to be 14.90 ± 0.98% in 120 min in pH 1.2 HCl solution and 28.65 ± 1.8% in 120 min from pH 6.8 buffer. The release of CAR from the CAR-SCS (F8) was found to be 89.85 ± 1.35 % in 120 min in pH 1.2 HCl solution and 94.4 ± 1.5 % in 120 min in pH 6.8 buffer. A high dissolution rate of the CAR-SCS (F8) compared to the CAR can be attributed to the formation of SCS, wherein the crystalline drug is suspended in the melted crystalline hydrophilic carrier. The presence of the crystalline hydrophilic carrier around CAR in the CAR-SCS enhances the polarity and decreases the interfacial tension between CAR and the dissolution medium, thus improving the dissolution rate and solubility. ^50^

### *Ex vivo* intestinal permeation studies

**Figure 16.**
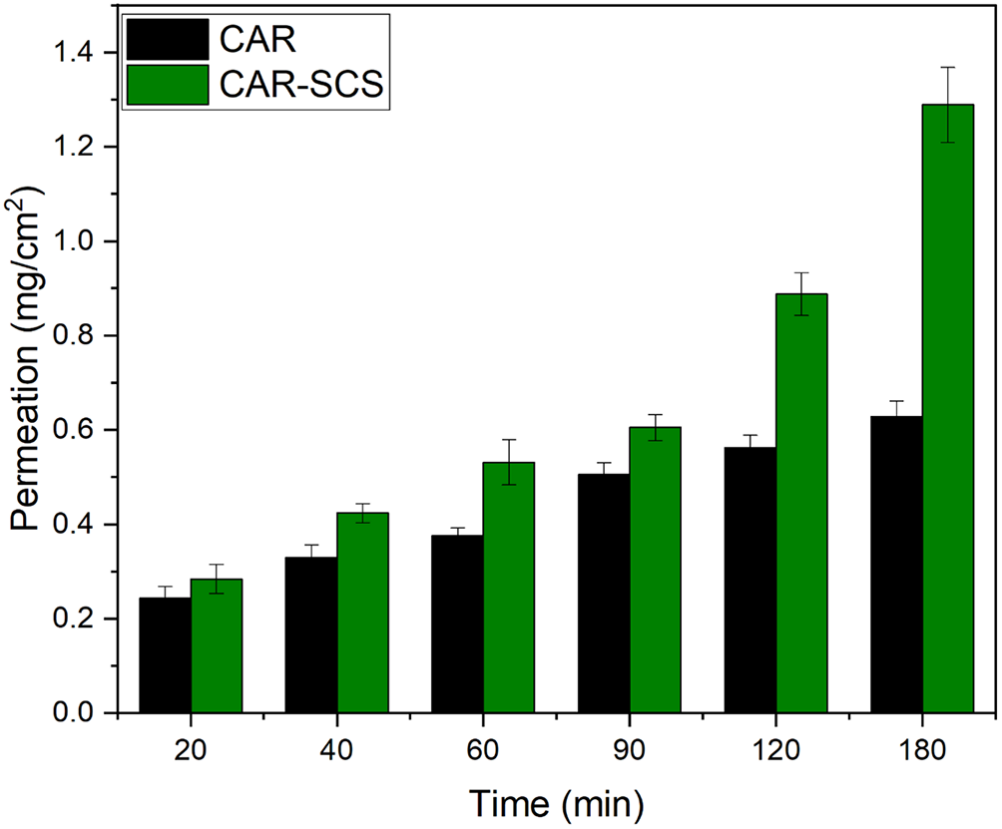
*Ex vivo* intestinal permeation profiles of Carvedilol (plain CAR) and Carvedilol-Solid crystal suspension (F8). Note: Data represented as mean± SD (n=3).

The permeation profile of the optimized CAR-SCS *via* the non-everted intestinal segment at different time intervals is depicted in Figure 16. The apparent permeability coefficient (P *app*) values for plain CAR and CAR-SCS (F8) formulation were found to be 0.066 cm/min and 0.122 cm/min. The P *app* for optimized CAR-SCS (F8) was 1.84 folds higher than plain CAR which could be possibly due to the higher permeation of the CAR-SCS (F8) formulation *via* the intestinal membrane owing to its better solubility and dissolution rate compared to the plain CAR. Drug absorption is a result of the capacity of the drug to diffuse through the lipophilic membrane of the intestine and its solubility in the aqueous milieu. Hence, the drug must be dissolved adequately to ensure higher permeation and bioavailability. ^53^ The higher permeation of the CAR-SCS (F8) compared to the CAR could be because of the higher solubility of CAR in the melted hydrophilic carrier mannitol which helps in wetting of the CAR particles facilitating the dissolution and permeation via intestinal membrane.

### *In vivo* pharmacokinetic (PK) study

**Figure 17.**
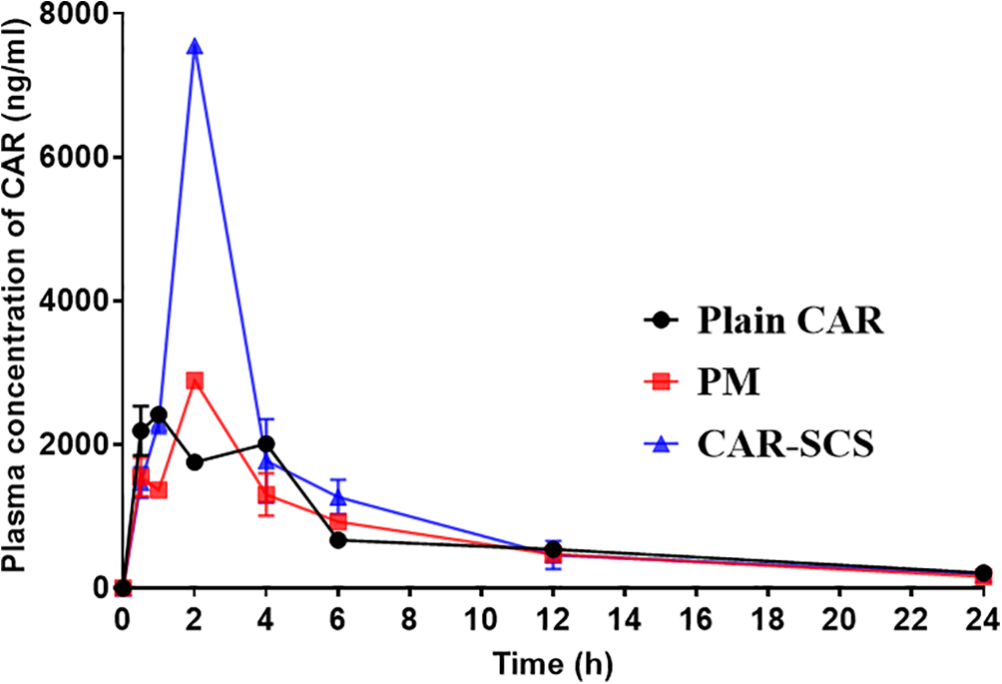
*In vivo* plasma concentration of Carvedilol, Carvedilol: mannitol Physical Mixture and Carvedilol-solid crystal suspension (F8). Note: Data represented as mean± SD (n=4).

The *in vivo* performance of CAR-SCS (F8) was evaluated and is represented in Figure 17. Compared to CAR alone and PM, CAR-SCS showed improved *in vivo* PK profile. Figure 17 indicates the plasma concentration-time profile of the CAR, PM and the CAR-SCS (F8). The Cmax and tmax after administering single dose of 40 mg/kg of CAR were 2459.95 ± 35.00 ng/mL and 0.75 ± 0.353 h respectively. The C _max_ of PM and CAR-SCS (F8) was enhanced 1.176-fold and 3.07-fold respectively in comparison to the C _max_ of plain CAR. Hence, CAR-SCS indicated an increase in the C _max_ and thus higher bioavailability of the CAR-SCS (F8) formulation compared to the plain CAR and the PM. The difference in the tmax and Cmax values for the CAR, PM and CAR-SCS (F8) was statistically significant ( *p* < 0.05). The t _1/2_ of CAR-SCS (F8) was 1.28 times higher than the plain CAR depicting that the CAR-SCS showed a prolonged residence time in the body compared to the plain CAR. The AUC _0-24_ for the CAR-SCS enhanced 1.50-fold than the plain CAR, indicating a noteworthy enhancement in the oral bioavailability of CAR in the CAR-SCS (F8) formulation. From the PK studies it was evident that the CAR-SCS (F8) formulation enhanced the plasma concentration, oral bioavailability and increased the residence time of the CAR in the body compared to the PM and plain CAR. The pharmacokinetic parameters are represented in Table 7. This enhanced oral bioavailability may be due to the (i) enhancement in the solubility, dissolution and permeation of the CAR in the CAR-SCS, (ii) enhanced passive diffusion of CAR *via* the intestinal membrane owing to the increased concentration gradient.

**Table 7.** Pharmacokinetic parameters of Carvedilol, Carvedilol-mannitol PM and optimized Carvedilol-Solid crystal suspension (F8).

PK parameters	Pure CAR suspension	PM	CAR-SCS (F8)
Tmax (h)	0.75 ± 0.353	2.00 ± 0.0	2.00 ± 0.0
C max (ng/mL)	2459.95 ± 35.00	2894.40 ± 51.47	7557.4 ± 18.52
AUC _0-24_ (h*ng/mL)	21174.80 ± 452.30	19423.07 ± 1099.6	30916.21 ± 2399.2
AUC _0-∞_ (h*ng/mL)	18405.81 ± 16.09	17628.53 ±770.18	27731.98 ± 3971.8
T _1/2_ (h)	8.945 ± 0.500	7.751 ± 0.709	11.52 ± 5.88
K _el_ (h ^-1^)	0.0775 ± 0.005	0.096 ±0.008	0.069 ± 0.035

### Stability studies

**Figure 18.**
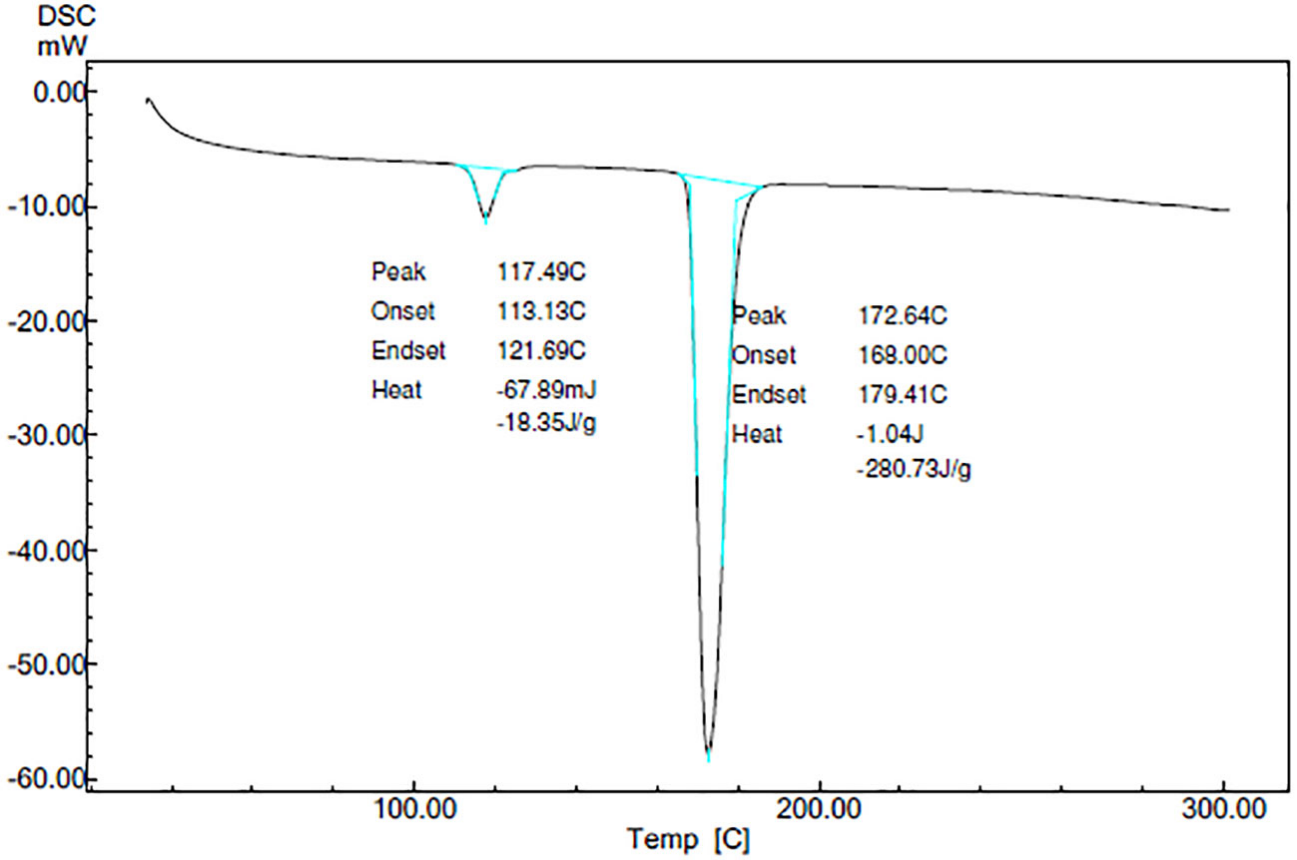
DSC Spectra of optimized Carvedilol -solid crystal suspension (F8) at the end of 3 months of stability period.

The stability studies were performed on the CAR-SCS (F8) formulation at accelerated stability conditions (40°C/75% RH) for 3 months (Thermolab stability chamber, 500 L). The stability studies revealed that the CAR-SCS (F8) was stable for a period of 3 months. DSC studies revealed the crystallinity of the CAR-SCS (F8) even after 3 months of storage at accelerated stability conditions. The same DSC pattern was observed throughout the stability period ( Figure 18). The % drug content for the CAR-SCS (F8) after a period of 1 month, 2 months and 3 months was found to be 77.73 ± 0.98 %, 77.70 ± 1.00 %, and 77.50 ± 1.00 %. The results indicated that the CAR-SCS (F8) was stable for three months with no change in its appearance and % drug content.

## Conclusions

Solid crystal suspensions offer a unique way for the solubility enhancement for the poorly aqueous soluble drugs. There are no reports on the preparation of SCS by a TSP process that utilizes co-rotating twin screws with different heating zones within the barrel. The solubility of CAR is affected by the high crystal lattice energy, and the solubility of CAR was considerably enhanced with mannitol. The CAR-SCS (F8) optimized by Design Expert software, consisted of a 20:80 ratio of CAR:mannitol processed at the kneading zone temperatures of 120°C and 100 rpm screw speed. The CAR:mannitol ratios and the kneading zone temperature for the optimized solution obtained from the Design Expert software was similar to one amongst the four best solutions suggested by the *in-silico* MD studies. Also, the stability of the optimum CAR-SCS with CAR:mannitol ratio of 20:80, and a kneading zone temperature of 120°C was assessed using *in-silico* MD studies. The optimized CAR-SCS, prepared by utilizing a 20:80 ratio of CAR:mannitol at the kneading zone temperatures of 120°C, showed a higher hydrophilicity and lesser hydrophobicity according to the *in silico* MD studies. Higher solubility for the optimal CAR-SCS (F8) could be attributed to higher hydrophilicity of the CAR-SCS (F8) as evident from the *in silico* MD studies. DSC studies revealed the presence of CAR in the stable crystalline form in the CAR-SCS (F8). The *in vitro* dissolution studies for the optimized formulation (F8) revealed a 6.03 times enhancement in dissolution rate compared to plain CAR in pH 1.2 HCl solution and a 3.40 times enhancement in pH 6.8 buffer. The results of the permeation studies illustrated that the P *app* for optimized F8 formulation was higher than plain CAR. The cumulative amount of drug permeated from the plain CAR and CAR-SCS (F8) at the end of 180 min was 47.67% and 98.85% respectively. The *in vivo* pharmacokinetic study indicated a 3.07-fold enhancement in the C _max_ and 1.28-fold enhancement in t _1/2_ of CAR-SCS (F8) than the plain CAR) compared to the plain CAR depicting a significant enhancement in the oral bioavailability and prolonged residence time in the body respectively. The present platform technology and expertise involving co-rotating TSP instrument with different heating zones used in the development of CAR-SCS could be applied to various drug candidates with poor solubility[1].

## Data Availability

Carvedilol data is available from: (no date) National Center for Biotechnology Information. PubChem Compound Database. Available at: https://pubchem.ncbi.nlm.nih.gov/compound/carvedilol (Accessed: 09 September 2023). ^56^ Mannitol data is available from: (no date) National Center for Biotechnology Information. PubChem Compound Database. Available at: https://pubchem.ncbi.nlm.nih.gov/compound/MANNITOL (Accessed: 09 September 2023). ^56^ FigShare. Raw data, DOI: https://doi.org/10.6084/m9.figshare.23613693. ^57^ This project contains the underlying data:
a. The *in vivo* pharmacokinetic data for the CAR, PM and the CAR-SCS (F8) b. The ANOVA data generated by the Design Expert software c. FTIR raw data for (i) plain CAR (ii) Mannitol (iii) PM and (iv) CAR-SCS (F8) d. DSC raw data for (i) plain CAR (ii) Mannitol (iii) PM and (iv) CAR-SCS (F8) and e. XRD raw data for (i) plain CAR (ii) Mannitol (iii) PM and (iv) CAR-SCS (F8). Data is available under the terms of Creative Commons Zero “No rights reserved” data waiver (CC0 1.0 Public domain dedication). FigShare: Untitled Item. https://doi.org/10.6084/m9.figshare.23936067. ^58^ This project contains the following ARRIVE guideline files:
- ARRIVE09.09.2023.pdf
