# Supplementary material for: Co-rotating twin screw process for continuous manufacturing of solid crystal suspension: A promising strategy to enhance the solubility, permeation and oral bioavailability of Carvedilol
Source: F1000Res. 2024 May 22;12:1438. Originally published 2023 Nov 6. [Version 3] doi: 10.12688/f1000research.139228.3 (PMC11109692; doi:10.12688/f1000research.139228.3)
Supplement: Peer review history

# Reviewer report: Reviewer response for version 2

**DOI:** 10.5256/f1000research.162978.r273222

**Published:** 2024-05-09

**Authors:** Kensaku Matsunami

**Affiliations:** 1 Ghent University, Ghent, Belgium

No further comments.

Is the work clearly and accurately presented and does it cite the current literature?

Partly

If applicable, is the statistical analysis and its interpretation appropriate?

Partly

Are all the source data underlying the results available to ensure full reproducibility?

Yes

Is the study design appropriate and is the work technically sound?

Yes

Are the conclusions drawn adequately supported by the results?

Partly

Are sufficient details of methods and analysis provided to allow replication by others?

Yes

Reviewer Expertise:

Pharmaceutical engineering, process systems engineering, experimental design, data analysis, mechanistic modeling

I confirm that I have read this submission and believe that I have an appropriate level of expertise to confirm that it is of an acceptable scientific standard.

# Response

**Published:** 2024-05-09

**Authors:** Srinivas Mutalik

**Affiliations:** Pharmaceutics, Manipal Academy of Higher Education, Manipal, Karnataka, India

Dear Sir/ Madam,

 We sincerely thank the reviewer for going through the article and providing constructive comments, which helped us present our manuscript much better. We revised the article meticulously according to the comments. We also thank the reviewer for Approving the revised manuscript for publication in F1000Research.

# Reviewer report: Reviewer response for version 2

**DOI:** 10.5256/f1000research.162978.r258746

**Published:** 2024-04-19

**Authors:** Pavan Kumar Nukala

**Affiliations:** 1 St John's University, New York, New York, USA

1. Include comment about the solid state of CAR in final formulation F8 (Amorphous or Crystalline) in the abstract.
2. The authors have done a great work and presented well in their manuscript. However, to improve the clarity for readers and to enhance the understanding of readers, kindly elaborate the figure legends in description. For example in Fig 5, it’s hard to identify the individual thermograms while reading the figure description.
3. In Fig 13, indicate in the figure about the media in which test has been conducted. Modify the Y axis title by addition of media information.
4. CAR exhibits pH dependent solubility and its vital to discuss this while explaining results.
5. It would have been a better study if the authors had performed a comparative dissolution against marketed formulation.
6. Did the authors try to understand the interaction of two parameters statistically at the same time affecting the CPP?
7. In Figure 7, circle the sharp characteristic peak of CAR at 3342.33 cm-1 in the formulation to highlight the decreased intensity.
8. Its crucial to discuss all the physicochemical properties of CAR in the introduction.
9. I agree that the authors have conducted *In vitro* and *In vivo* studies, it would have added more strength to the results if the experiments were conducted using the marketed formulation as well to understand the superiority of F8 formulation.
10. Usually, the ACC stability studies will be conducted until 6M timepoint. Any specific reason for testing the stability samples only at 3M timepoint?
11. Kindly edit the sentence 'good stability...' in the abstract to more technical terms.
12. Kindly cite the recent literature from the past 5 years (2019-2024) to support the results and discussion.

Is the work clearly and accurately presented and does it cite the current literature?

Partly

If applicable, is the statistical analysis and its interpretation appropriate?

I cannot comment. A qualified statistician is required.

Are all the source data underlying the results available to ensure full reproducibility?

Yes

Is the study design appropriate and is the work technically sound?

Yes

Are the conclusions drawn adequately supported by the results?

Partly

Are sufficient details of methods and analysis provided to allow replication by others?

Partly

Reviewer Expertise:

Amorphous solid dispersions, Solubility enhancement,  Modified release formulations.

I confirm that I have read this submission and believe that I have an appropriate level of expertise to confirm that it is of an acceptable scientific standard, however I have significant reservations, as outlined above.

# Response

**Published:** 2024-05-06

**Authors:** Srinivas Mutalik

**Affiliations:** Pharmaceutics, Manipal Academy of Higher Education, Manipal, Karnataka, India

The authors are grateful for the constructive comments of Reviewer 3. We have thoroughly revised the manuscript as per the reviewer comments.

 1. Include comment about the solid state of CAR in final formulation F8 (Amorphous or Crystalline) in the abstract.

**Ans: **The abstract has been modified according to the reviewer comments

 2. The authors have done a great work and presented well in their manuscript. However, to improve the clarity for readers and to enhance the understanding of readers, kindly elaborate the figure legends in description. For example in Fig 5, it’s hard to identify the individual thermograms while reading the figure description.

**Ans: **The Figure legends have been changed as per reviewer’s suggestions. Figure legends have been modified in Fig 5, Fig 7, Fig 8 and Fig 11.

 3. In Fig 13, indicate in the figure about the media in which test has been conducted. Modify the Y axis title by addition of media information.

**Ans**: We have modified Fig 13 as per the reviewer suggestions with the media name as pH 6.8 buffer in the manuscript.

 5. ****CAR exhibits pH dependent solubility and its vital to discuss this while explaining results.

**Ans:** The sentence has been included in the solubility results as per the reviewer suggestions

 4. It would have been a better study if the authors had performed a comparative dissolution against marketed formulation.

**Ans:** We could not perform the comparison with the marketed formulation. However, we have compared the results of the CAR-SCS (F8) formulation with the pure drug and CAR-SCS (F8) showed enhanced solubility compared to the plain drug. Comparison with the marketed formulation could be considered for the future scope of the study.

 6. Did the authors try to understand the interaction of two parameters statistically at the same time affecting the CPP?

**Ans: ** A linear model was selected for the analysis, hence we did not study the interaction of two parameters at the same time affecting the CPP.

 7. In Figure 7, circle the sharp characteristic peak of CAR at 3342.33 cm-1 in the formulation to highlight the decreased intensity.

**Ans:** In Figure 7, the peak has been already highlighted with a dotted line which is evident in all three, Carvedilol, CAR-SCS (F8) and Physical mixture.

 8. It's crucial to discuss all the physicochemical properties of CAR in the introduction.

**Ans:** As per the reviewer suggestions, the physicochemical properties of CAR have been included in the introduction.

 9. I agree that the authors have conducted In vitro and In vivo studies, it would have added more strength to the results if the experiments were conducted using the marketed formulation as well to understand the superiority of F8 formulation.

**Ans: **We did not perform the comparison with the marketed formulation. However, we have compared the results of the CAR-SCS (F8) formulation with the pure drug and CAR-SCS (F8) showed enhanced solubility, in vivo and in vitro activity compared to the plain drug. Comparison with the marketed formulation could be considered for the future scope of the study.

 10. Usually, the ACC stability studies will be conducted until 6M timepoint. Any specific reason for testing the stability samples only at 3M timepoint?

**Ans:** In our study we have only conducted stability studies for the period of 3 months. The product CAR-SCS (F8) was very stable for 3 months period with respect to the appearance, % in vitro drug content and crystallinity. However, long term stability studies could be considered in the future scope of the study.

 11. Kindly edit the sentence 'good stability...' in the abstract to more technical terms.

**Ans: **The term good stability has been replaced with the words satisfactory solubility in the abstract as suggested by the reviewer in the manuscript.

 12. Kindly cite the recent literature from the past 5 years (2019-2024) to support the results and discussion.

**Ans:** The recent literature has been cited as per the reviewer comments.

# Reviewer report: Reviewer response for version 2

**DOI:** 10.5256/f1000research.162978.r258747

**Published:** 2024-04-02

**Authors:** Dhaval Mori

**Affiliations:** 1 B.K. Mody Government Pharmacy College Polytechnic, Rajkot, India

The present investigation describes the formation of solid crystal suspension for improving the dissolution of the drug carvadiol. The solid crystal suspension was prepared by hot melt extrusion method using mannitol and sorbitol as crystalline carrier. The authors have optimized the process parameters such as screw rotation speed, temperature of kneading zone and ratio of drug to carrier using DoE. The prepared product was evaluated using analytical tool such as PXRD, FTIR, DSC , Raman spectroscopy etc. The saturation solubility and in vitro dissolution was also performed to check the effectiveness of the method. Ex vivo permeability study and in vivo pharmacokinetic study was also performed for the product.

 Overall the products is thoroughly characterized and evaluated however following are some of the technical comments needs to be justified before further processing

  

1. The screw design is at the heart of twin screw extruder operation. The authors required to give screw design for the mentioned experiments.
2. The so called solid crystal suspension are first generation solid dispersion uses crystalline material with better solubility to improve the dissolution of poorly soluble drugs. The mechanism includes better wettability of the drug. In the introduction section the authors have mentioned wetting and particle size reduction for improving the dissolution and solubility. The improvement in the dissolution with respected to reduced particle size can be explained by noyes whitney equation. Please explain how solubility can be improved by reducing particle size. Please explain with required references.
3. Page no 10, statical analysis “to increase with decreasing kneading zone temperatures and increasing screw rpm. This might be because, as the temperature increases nearing to the melting point of the CAR, it forms a sticky mass with the hydrophilic mannitol due to over-melting of CAR ” However, according to DSC study the melting point of carvadiol is around 117 °C and processing temperature ranged between 120 °C to 140 °C so in all the experimental condition the temperature was above the melting point of the drug and the drug was expected to be in  liquid state so how increasing the temperature can lead to more sticky mass as all the temperature is above melting point only
4. In the same section the authors have mentioned that “TSP thereby resulting in drug loss leading to the lesser solubility and % yield of the resulting product.” The saturation solubility of different batches are performed by taking equivalent mass of the drug so of the drug is lost during the process due to sticking and all than the % product yield can be less but how it can effect the solubility as the it needs equivalent mass to be added in the dissolution media to measure.
5. In the same section the authors have mentioned “Solubility was slightly affected by the CAR:mannitol ratio” Please explain the meaning of slightly ? how much to be consider.
6. The results mentioned in the DSC, FTIR and PXRD are contradictory. In the DSC section the authors have mentioned that “the DSC results for the CAR-SCS (F8) confirm the crystallinity of the CAR-SCS (F8) and indicate that both CAR and mannitol occur as distinct crystalline phases” Means both the material retained their structure and there is no formation of new phase as no new peak was generated in the DSC. However, in the FTIR and PXRD the authors have concluded formation of new phases “1:1 physical mixture of CAR and mannitol, was found to show decreased intensity in the CAR-SCS (F8) indicating the new product formation.” “ . 2θ values for the CAR-SCS (F8) were different from the starting materials. Also, the number of peaks, intensity and the positions of the diffraction peaks for CAR-SCS (F8) were different from the starting materials and PM indicating the interaction between CAR and mannitol to form CAR-SCS (F8)” The authors are requested to remove this discrepancy and write congruent conclusion. The authors need to justify with suitable reference that if there should be reaction between the drug and crystalline carrier or there should be not.

Is the work clearly and accurately presented and does it cite the current literature?

Partly

If applicable, is the statistical analysis and its interpretation appropriate?

Partly

Are all the source data underlying the results available to ensure full reproducibility?

Yes

Is the study design appropriate and is the work technically sound?

Partly

Are the conclusions drawn adequately supported by the results?

Partly

Are sufficient details of methods and analysis provided to allow replication by others?

Yes

Reviewer Expertise:

oral solid dosage form, salt formation, amorphous solid dispersion

I confirm that I have read this submission and believe that I have an appropriate level of expertise to confirm that it is of an acceptable scientific standard, however I have significant reservations, as outlined above.

# Response

**Published:** 2024-04-09

**Authors:** Srinivas Mutalik

**Affiliations:** Pharmaceutics, Manipal Academy of Higher Education, Manipal, Karnataka, India

Dear Reviewer,

 We thank you for the constructive and thoughtful review of our manuscript. We appreciate your time, valuable feedback and suggestions. We have carefully revised manuscript according to your comments and made revisions. Here, is our detailed response to the comments:

 1. The screw design is at the heart of twin screw extruder operation. The authors required to give screw design for the mentioned experiments.

**Ans:** The details regarding screw sections and design have been added in the manuscript in the section, “Selection of crystalline polyol for the preparation of solid crystal suspension of carvedilol”.

 2. The so called solid crystal suspension are first generation solid dispersion uses crystalline material with better solubility to improve the dissolution of poorly soluble drugs. The mechanism includes better wettability of the drug. In the introduction section the authors have mentioned wetting and particle size reduction for improving the dissolution and solubility. The improvement in the dissolution with respected to reduced particle size can be explained by Noyes whitney equation. Please explain how solubility can be improved by reducing particle size. Please explain with required references

**Ans:** The improvement in the solubility and dissolution with respect to reduced particle size have been added along with the references on page no. 4 in the manuscript.

 3. Page no 10, statical analysis “to increase with decreasing kneading zone temperatures and increasing screw rpm. This might be because, as the temperature increases nearing to the melting point of the CAR, it forms a sticky mass with the hydrophilic mannitol due to over-melting of CAR ” However, according to DSC study the melting point of carvadilol is around 117 °C and processing temperature ranged between 120 °C to 140 °C so in all the experimental condition the temperature was above the melting point of the drug and the drug was expected to be in  liquid state so how increasing the temperature can lead to more sticky mass as all the temperature is above melting point only.

**Ans:** The sentence has been replaced in the manuscript. It is possible that at temperatures more than 120°C, the interactions between CAR and mannitol could be altered or changes in the thermal characteristics, physical properties or viscosity of the materials used or some interplay of factors which need investigation.

 4. In the same section the authors have mentioned that “TSP thereby resulting in drug loss leading to the lesser solubility and % yield of the resulting product.” The saturation solubility of different batches are performed by taking equivalent mass of the drug so of the drug is lost during the process due to sticking and all than the % product yield can be less but how it can effect the solubility as the it needs equivalent mass to be added in the dissolution media to measure.

**Ans:** Suggested changes to the statement have been made. The sentence has been replaced in the manuscript.

 5. In the same section the authors have mentioned “Solubility was slightly affected by the CAR:mannitol ratio” Please explain the meaning of slightly? how much to be consider.

**Ans:** The sentence has been modified as “Solubility was affected by the CAR:mannitol ratio and showed a decrease with the increase in CAR:mannitol ratio“.

 6. The results mentioned in the DSC, FTIR and PXRD are contradictory. In the DSC section the authors have mentioned that “the DSC results for the CAR-SCS (F8) confirm the crystallinity of the CAR-SCS (F8) and indicate that both CAR and mannitol occur as distinct crystalline phases” Means both the material retained their structure and there is no formation of new phase as no new peak was generated in the DSC. However, in the FTIR and PXRD the authors have concluded formation of new phases “1:1 physical mixture of CAR and mannitol, was found to show decreased intensity in the CAR-SCS (F8) indicating the new product formation.” “ . 2θ values for the CAR-SCS (F8) were different from the starting materials. Also, the number of peaks, intensity and the positions of the diffraction peaks for CAR-SCS (F8) were different from the starting materials and PM indicating the interaction between CAR and mannitol to form CAR-SCS (F8)” The authors are requested to remove this discrepancy and write congruent conclusion. The authors need to justify with suitable reference that if there should be reaction between the drug and crystalline carrier or there should be not.

**Ans:** The suggested changes have been made in the manuscript. In CAR-SCS (F8), CAR and mannitol solely occur as distinct phases and do not interact as per DSC studies. CAR-SCS (F8) showed the presence of FTIR, DSC and XRD peaks characteristic to both CAR and mannitol. Changes have been made in the manuscript with respect to the conclusion part on page no 18 and 19.

# Reviewer report: Reviewer response for version 1

**DOI:** 10.5256/f1000research.152488.r227292

**Published:** 2024-01-18

**Authors:** Kensaku Matsunami

**Affiliations:** 1 Ghent University, Ghent, Belgium

This paper presents the application of the co-rotating twin-screw process toward the enhancement of the solubility, permeation, and oral bioavailability of Carvedilol. The authors have worked on a large variety of experimental studies (e.g., in-vivo and ex-vivo studies) as well as simulations. The paper is indeed interesting.

 On the other hand, the paper does not show sufficient discussion and conclusion. It seems like the authors have performed different approaches and shown all results. To be approved as a research article, the authors need to revise the discussion and the conclusion sessions to make sure "what each result means in terms of the objective of the study" and "what is the general conclusion obtained through different types of studies."

 While the reviewer understands the value of MD or in-silico approaches, it is not clear why in-silico approaches are necessary for your study. The introduction should be improved so that the readers can understand the necessity of MD studies.

 The followings are minor comments.

**Table 1**: Better to add units on the top (especially for temperature).

**Formulation of Solid crystal suspension of Carvedilol (CAR-SCS) by TSP using BBD: **the authors wrote "TSP instrument parameters like kneading zone temperature, screw rpm, and formulation-related parameter (CAR: mannitol ratio)," but were there any parameters the authors assessed? If not, it is better to clarify that they are "the" parameters the authors considered.

**Results- Statistical analysis and optimization using BBD: **The results of ANOVA analysis are not clear. Normally, ANOVA results tell which main effects and interactions are statistically significant. However, no value is shown for each factor.

 The authors did only one trial for the validation. The reviewer wonders if it is not sufficient to validate the model and the authors do either more validation or other analysis to prove the accuracy of the model.

Is the work clearly and accurately presented and does it cite the current literature?

Partly

If applicable, is the statistical analysis and its interpretation appropriate?

Partly

Are all the source data underlying the results available to ensure full reproducibility?

Yes

Is the study design appropriate and is the work technically sound?

Yes

Are the conclusions drawn adequately supported by the results?

Partly

Are sufficient details of methods and analysis provided to allow replication by others?

Yes

Reviewer Expertise:

Pharmaceutical engineering, process systems engineering, experimental design, data analysis, mechanistic modeling

I confirm that I have read this submission and believe that I have an appropriate level of expertise to confirm that it is of an acceptable scientific standard, however I have significant reservations, as outlined above.

# Response

**Published:** 2024-02-20

**Authors:** Srinivas Mutalik

**Affiliations:** Pharmaceutics, Manipal Academy of Higher Education, Manipal, Karnataka, India

Dear Sir/ Madam,

 We thank you for the thoughtful review of our manuscript and constructive comments. We appreciate your valuable feedback and suggestions. We have carefully addressed your comments and made revisions. Here, is our detailed response to the comments:

- To be approved as a research article, the authors need to revise the discussion and the conclusion sessions to make sure "what each result means in terms of the objective of the study" and "what is the general conclusion obtained through different types of studies."

**Reply:** We have made changes in terms of what every result means and changes in the conclusion regarding the different studies.

  

- it is not clear why in-silico approaches are necessary for your study. The introduction should be improved so that the readers can understand the necessity of MD studies.

**Reply**:  We have included *in-silico* molecular dynamics (MD) aspects for studying the interactions between the CAR and mannitol in different ratios of CAR: mannitol (20:80, 35:65, and 50:50) employed in the study at various kneading zone temperatures (120 °C, 130 °C, and 140 °C) to predict the optimal ratios for preparing CAR-SCS formulations. This information is given in Introduction section as suggested.

**Minor Comments**

- **Table 1**: Better to add units on the top (especially for temperature).

**Reply**- The units for the temperature (°C) have been added.

- **Formulation of Solid crystal suspension of Carvedilol (CAR-SCS) by TSP using BBD: **the authors wrote "TSP instrument parameters like kneading zone temperature, screw rpm, and formulation-related parameter (CAR: mannitol ratio)," but were there any parameters the authors assessed? If not, it is better to clarify that they are "the" parameters the authors considered.

**           Reply-**The parameters screw rpm and kneading zone temperature was selected on the basis of the preliminary trials conducted. However, we considered varying CAR:mannitol ratio as a formulation related parameter to check whether varying the mannitol concentrations could have impact on the CAR-SCS **. **This has been mentioned in the Experimental design section.

- **Results- Statistical analysis and optimization using BBD: **The results of ANOVA   analysis are not clear. Normally, ANOVA results tell which main effects and interactions are statistically significant. However, no value has been shown for each factor.

**Reply**- The details of ANOVA data and the value for each factor has been included the raw data uploaded with the DOI link https://doi.org/10.6084/m9.figshare.23613693.  The suggested changes have been made in the manuscript. The ANOVA details have been included in the ‘Statistical analysis and optimization using BBD’ section.

 We thank you for your valuable feedback, expertise and time in reviewing our manuscript and we believe that the revisions have improved the manuscript significantly.
